# Molecular Dynamics Revealing a Detour-Forward Release Mechanism of Tacrine: Implication for the Specific Binding Characteristics in Butyrylcholinesterase

**DOI:** 10.3389/fchem.2020.00730

**Published:** 2020-08-25

**Authors:** Zhiyang Zhang, Fangfang Fan, Wen Luo, Yuan Zhao, Chaojie Wang

**Affiliations:** ^1^The Key Laboratory of Natural Medicine and Immuno-Engineering, Henan University, Kaifeng, China; ^2^School of Biological and Chemical Engineering, Zhejiang University of Science and Technology, Hangzhou, China

**Keywords:** Alzheimer's disease, tacrine, butyrylcholinesterase, release mechanism molecular dynamics, MM/GBSA, umbrella sampling

## Abstract

Butyrylcholinesterase (BChE) is a non-specific enzyme with clinical pharmacological and toxicological significance, which was a renewed interest as therapeutic target in Alzheimer's disease (AD) nowadays. Here, all-atom molecular dynamics simulations of butyrylcholinesterase with tacrine complex were designed to characterize inhibitor binding modes, strengths, and the hydrogen-bond dependent non-covalent release mechanism. Four possible release channels were identified, and the most favorable channel was determined by random acceleration molecular dynamics molecular dynamics (RAMD MD) simulations. The thermodynamic and dynamic properties as well as the corresponding Detour-forward delivery mechanism were determined according to the classical molecular dynamics (MD) simulations accompanied with umbrella sampling. The free energy barrier of the tacrine release process for the most beneficial pathway is about 10.95 kcal/mol, which is related to the non-covalent interactions from the surrounding residues, revealing the specific binding characteristics in the active site. The residues including Asp70, Ser79, Trp82, Gly116, Thr120, Tyr332, and His438 were identified to play major roles in the stabilization of tacrine in the pocket of BChE, where hydrogen bonding and π-π interactions are significant factors. Tyr332 and Asp70, which act as gate keepers, play crucial roles in the substrate delivery. The present results provide a basic understanding for the ligand transport mechanism depending on the BChE enzymatic environment, which is useful for the design of BChE inhibitors in the future.

## Introduction

Alzheimer's disease (AD), a progressive neurodegenerative disorder, is characterized by generalized dementia such as memory impairment, aphasia, apraxia, visual spatial skill impairment, executive dysfunction, etc. (Tschanz and Andersen, 2011). With the aging of the population, age-dependent neurodegenerative disorders have become a serious medical problem in modern society. Thus, tremendous material and financial resources are gradually devoted into treatment of AD. Whereas, the etiology of AD is not completely understood due to its being complex and multifactorial, low level of acetylcholine (ACh), β-amyloid (Aβ) aggregation, tau-protein hyperphosphorylation, oxidative stress, etc. (Grundke-Iqbal et al., 1986; Talesa, 2001; Hardy, 2009; Rosini et al., 2014) all have been correlated at some extent to AD.

Cholinesterases (ChEs), including acetylcholinesterase (AChE) and butyrylcholinesterase (BChE), play important roles in cholinergic transmission by hydrolysis of the neurotransmitter acetylcholine (ACh) (Masson et al., 1999). Many studies have found that AD is related to the decrease in acetylcholine (ACh), a neurotransmitter in the brain (Anand and Singh, 2013a; Sun et al., 2015; Zhu et al., 2017). Actually, the shortfall in cholinergic activity affects the activity of AChE and BChE, both of which regulate acetylcholine in the human brain (Giacobini, 2003). It is worth noting that the level of AChE in the brain declines to 55–67%, while BChE increases to 120% of normal levels during the progressed AD, indicating that BChE plays a critical role for ACh hydrolysis in the late stage of AD (Greig et al., 2001; Mushtaq et al., 2014). Hence, the BChE is considered to be a potentially useful target (Giacobini, 2003; Hartmann et al., 2007; Nordberg et al., 2013). The importance of BChE has also been demonstrated by the AChE knockout mice model, in which BChE compensates for the lack of AChE, maintaining normal cholinergic pathways in AChE nullizygous animals (Xie et al., 2000; Mesulam M. M. et al., 2002). It has been discovered that BChE can replace AChE to hydrolyze ACh in the presence of a specific AChE inhibitor in the human brain (Mesulam M. et al., 2002). In recent years, increasing the level of ACh in the brain by inhibiting the biological activity of AChE is a significant therapeutic approach for the treatment of AD. Accordingly, inhibition of BChE can be viewed as an alternative for the restoration of cholinergic activity and improvement of cognitive performance for patients with AD (Mushtaq et al., 2014).

**Figure 1:**
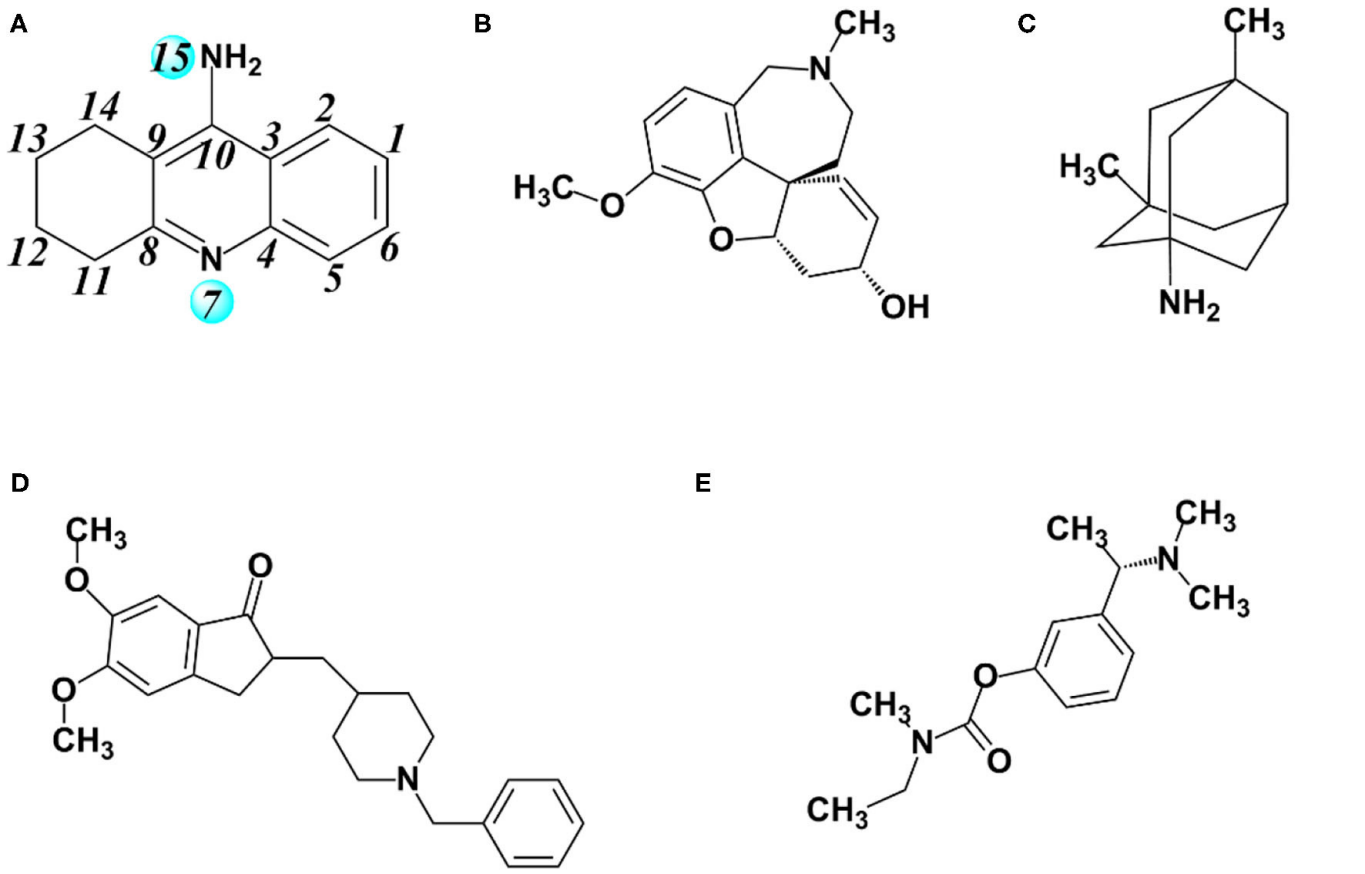
Major drugs approved by the US Food and Drug Administration (FDA) for treatment of Alzheimer's disease (AD). **(A)** Tacrine, **(B)** galantamine, **(C)** memantine, **(D)** donepezil, and **(E)** rivastigmine.

**Figure 2:**
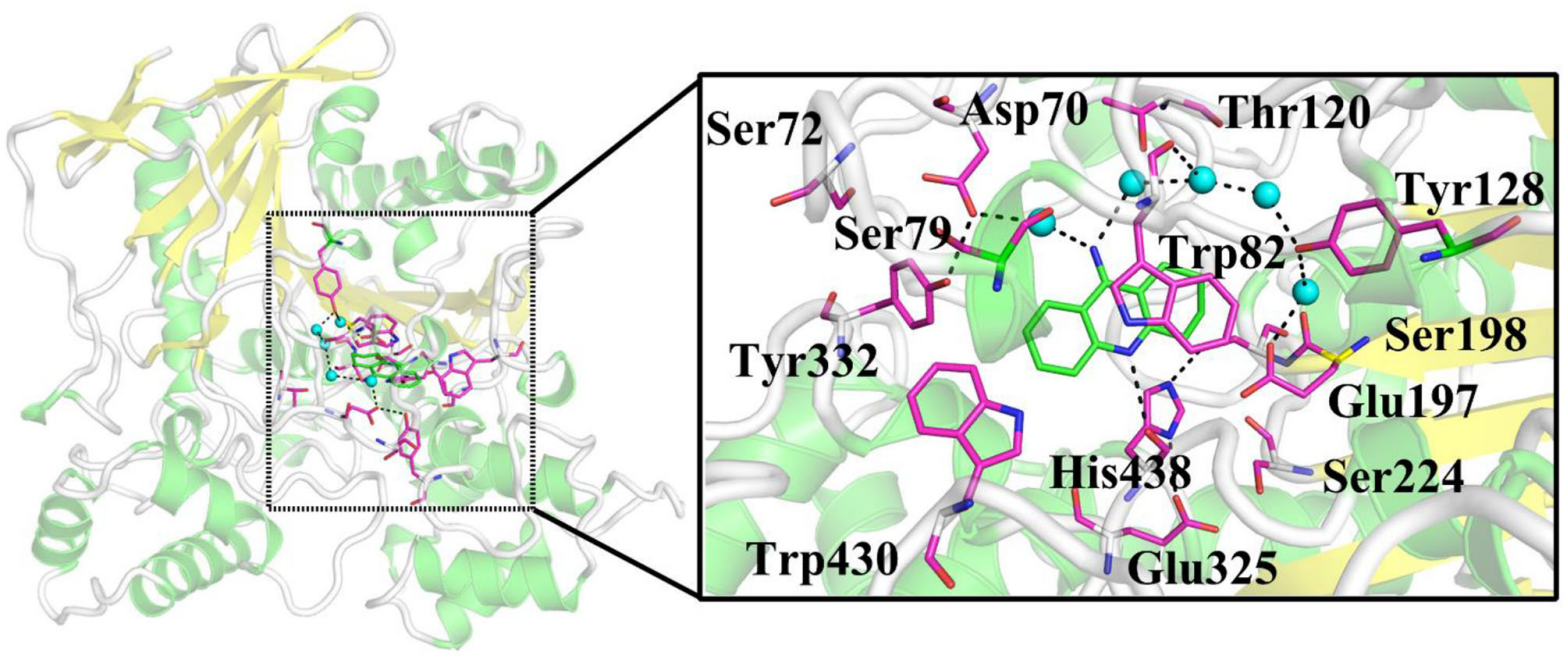
X-ray crystal structure of BChE complexed with tacrine (PDB ID: 4BDS). Key residues and ligand are represented as sticks, and waters are shown as turquoise spheres.

Nowadays, there are five anti-Alzheimer's disease drugs approved for clinical application (Figure 1), namely (a) tacrine, (b) galantamine, (c) memantine, (d) donepezil, and (e) rivastigmine, respectively. Among them, tacrine is a reversible inhibitor and a classical pharmacophore as the first generation of anti-AD agent, which was approved by the Food and Drug Administration (FDA) in 1993 (Summers, 2006). However, it was withdrawn (Qizilbash et al., 1999) due to its clinical shortcomings instigating hepatotoxicity (Lagadic-Gossmann et al., 1998). In spite of severe hepatotoxicity, tacrine is still considered as a good scaffold to design multitarget drugs for Alzheimer's disease (Bartolini and Marco-Contelles, 2019). Thus, the development of tacrine derivatives has drawn immense attention since tacrine is a classical pharmacophore and inhibits both AChE and BChE at micromolar scale (Sameem et al., 2017). Plenty of tacrine derivatives have been developed with the aim of improving inhibitory activity, eliminating toxicity, and enlarging properties as cholinesterase inhibitors (ChEIs) (Anand and Singh, 2013b). More recently, a new strategy has been proposed with the aim of designing compounds that can simultaneously target different targets that are closely related to AD. The compounds are referred to as multitarget-directed ligands (MTDLs), and tacrine is preferred in the designing of MTDLs (Bajda et al., 2011; Zhang et al., 2015; Lin et al., 2017; Chalupova et al., 2019). Moreover, the selective or non-selective inhibition of BChE may raise neuroprotective and disease-modifying effects (Greig et al., 2005). Therefore, the structural data of tacrine complex with BChE is extremely importance for developing the hopeful ChEIs, and it is recently resolved at 2.1 Å resolution (PDB ID: 4BDS) (Nachon et al., 2013). The X-ray cocrystal structure is shown in Figure 2. As we can see, key interactions mainly come from (1) the aromatic π-π stacking with Trp82; (2) hydrogen bonding between the N7 (as shown in Figure 1A) and the main chain carbonyl of His438; and (3) two hydrogen bonds between the amino group of tacrine and two water molecules in the surrounding water molecular network (Nachon et al., 2013).

The statistic crystal structure provides the intuitive structural model, while it is also significant to understand the interactions between BChE and tacrine, and several crucial questions are still open: (i) What is the specific contribution of key residues for tacrine binding in the active site? (ii) What are possible channels for the substrate tacrine delivery and which one is the most advantageous? (iii) How about the thermodynamic and dynamics properties and mechanisms during the tacrine release process? (iv) Which residues play crucial roles in the tacrine delivery process? Here, in order to clarify these issues, classical molecular dynamics simulations and extensive combined random acceleration molecular dynamics molecular dynamics (RAMD MD) simulations combined with the umbrella sampling technique have been performed. Such efforts can be served as reasonable initial stages to explore novel potential specific BChE inhibitors, which provide a molecular basis for designing new BChE inhibitors with better kinetic properties selectivity. The modeling of tacrine entrance into the active site is difficult to build for its uncertain positions outside the protein. Moreover, according to our previous studies, the process of ligand entrance into the active site is similarly reversible to the ligand release in a certain extent, especially for the possible pathways, delivery mechanisms, and conformation changes of protein (Zhao et al., 2016). Hence, here, we mainly focus on the tacrine release process from the active site to outside of BChE.

## Computational Methods and Details

### Setup of the Enzyme–Substrate Complex Model

**Figure 3:**
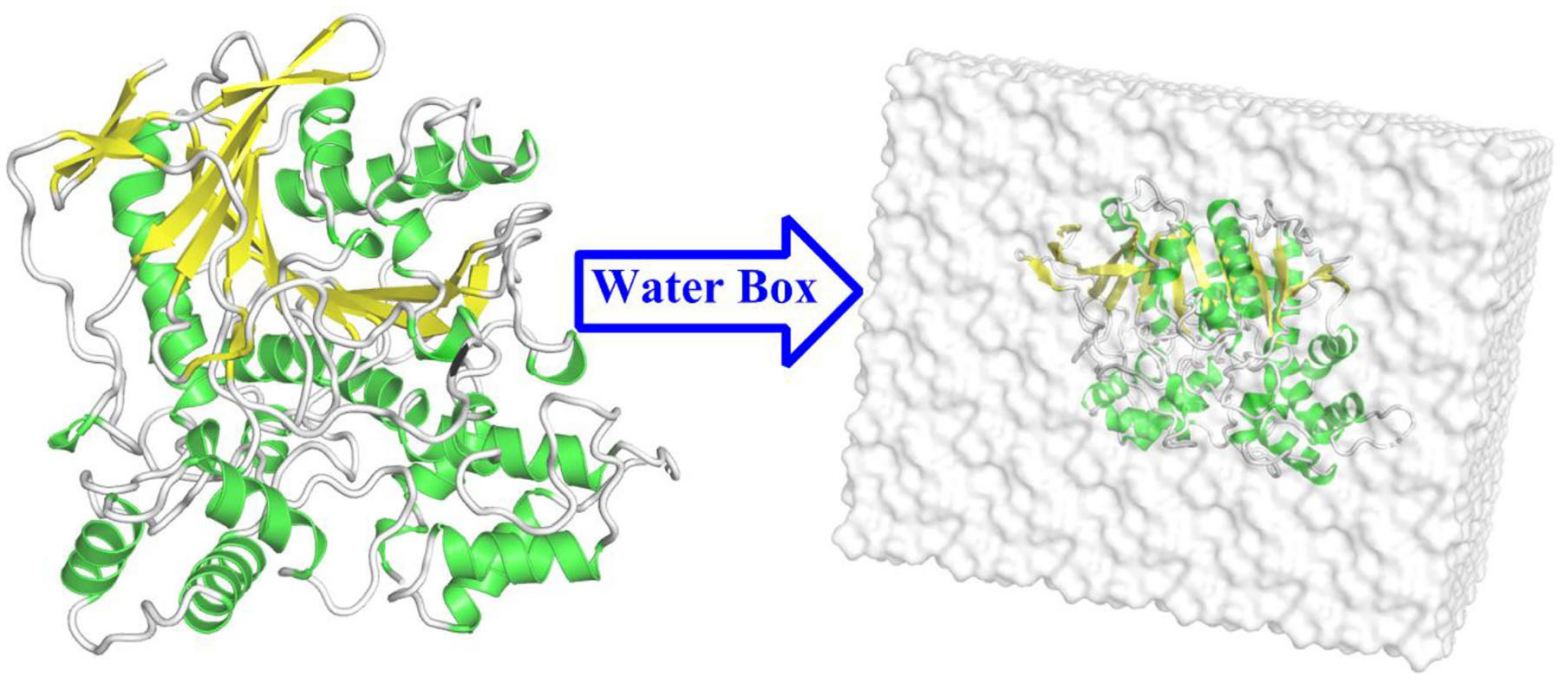
The structure of the butyrylcholinesterase (BChE) protein and the water box of the building model.

In the present study, the initial model for the simulations was acquired from the high-resolution crystal structures of BChE in complex with tacrine (PDB ID: 4BDS; resolution: 2.1 Å) (Nachon et al., 2013). The missing fragment of 378–379 residues was complemented by the biopolymer module in the (Sybyl-X 2.1, 2013) software. The proton state of ionizable residues was determined at pH 6.5 by PROPKA 3.0 (Rostkowski et al., 2011) as well as their surrounding environment. The partial atomic charges of tacrine were derived by the restrained electrostatic potential (RESP) (Cornell et al., 1993) charge at the HF/6-31G^*^ level with Gaussian03 software (Frisch et al., 2004). The protein and water molecules were described by the ff14SB force field (Maier et al., 2015; Shao and Zhu, 2018) and the TIP3P model, respectively (Jorgensen et al., 1983). The complex was dissolved in a cubic water box of 86 × 87 × 102 Å with a buffer distance of 10 Å on each side between the box wall and the nearest solute atoms, and then, the whole model box was neutralized with chlorine ions. The tleap modules in the AMBER 16 (Case et al., 2016) software was used to generate the topology parameters and initial coordinates of the whole system. The process of the model building is depicted in Figure 3.

### Molecular Mechanical Molecular Dynamics Simulations

In order to remove poor interactions between atoms, the whole system was minimized for 10,000 steps by using the steepest descent method, followed by a conjugate gradient method for another 10,000 steps minimization. Afterwards, the system was heated from 0 to 300 K gradually for 100 ps, and then, another 100 ps NPT MD simulations were carried out to relax the density to ~1.0 g/cm^3^. Finally, 100 ns classical MD simulations with NPT ensemble by using periodic boundary condition were performed. Langevin method was employed to maintain the temperature at 300 K (Pastor et al., 1988). Finally, 100 ns classical MD simulations with NPT ensemble by using periodic boundary condition were performed, which adopt the nearest mirror image principle. The 12 Å cutoff value was set to calculate the van der Waals force and the electrostatic interaction given the desired accuracy and computing costs, which has been successfully used in several other approximations even in bigger systems (Zhao et al., 2018; Fan et al., 2019). All of the bonds with hydrogen atoms in the model were constrained with the SHAKE algorithm (Ryckaert et al., 1977). The stability of the backbone of BChE was evaluated using root-mean-square deviation (RMSD) analysis. Then, the hydrogen bonds were analyzed using the last 10 ns trajectories of the MD simulations. The whole MD simulations were performed using the AMBER 16 package (Case et al., 2016).

### Molecular Mechanics/Generalized Born Surface Area

Molecular mechanics/generalized born surface area (MM/GBSA) (Kollman et al., 2000; Wang et al., 2001, 2006) has been widely applied to calculate the free energy for various protein–ligand (Kuhn and Kollman, 2000; Huo et al., 2002; Brown and Muchmore, 2006; Hou and Yu, 2007), protein–protein, and protein–peptide (Wang and Kollman, 2000; Gohlke and Case, 2004; Hou et al., 2006; She et al., 2019; Wei et al., 2019) complexes successfully. The binding free energy between the ligand and receptor was calculated with the following equation. Moreover, MM/GBSA allows for rigorous free energy decomposition into contributions originating from different groups of atoms or types of interaction (Gohlke et al., 2003; Hou et al., 2008, 2011).

$$\begin{array}{l} \begin{array}{c} \Delta {\text{G}}_{\text{binding}}={\Delta \text{G}}_{\text{complex}}-{\Delta \text{G}}_{\text{protein}}-{\Delta \text{G}}_{\text{ligand}} \end{array} \end{array}$$

where ΔG_complex_, ΔG_protein_, and ΔG_ligand_ stand for the free energies of complex, receptor, and ligand, respectively (Srivastava and Sastry, 2012; Munnaluri et al., 2015). The free energy also can be estimated as the sum of three terms:

$$\begin{array}{l} \begin{array}{c} \Delta {\text{G}}_{\text{binding}}={\Delta \text{E}}_{\text{mm}}+\;{\Delta \text{G}}_{\text{sol}}-\text{T}\Delta \text{S} \end{array} \end{array}$$

where ΔE_mm_ is the total gas phase energy expressed as the sum of the internal (int), electrostatic (ele), and van der Waals (vdW) energies:

$$\begin{array}{l} \begin{array}{c} {\Delta \text{E}}_{\text{mm}}={\Delta \text{E}}_{\text{int}}+\;{\Delta \text{E}}_{\text{ele}}+{\Delta \text{E}}_{\text{vdW}} \end{array} \end{array}$$

$$\begin{array}{l} \begin{array}{c} \Delta {\text{G}}_{\text{sol}}={\Delta \text{G}}_{\text{GB}}+\;{\Delta \text{G}}_{\text{SA}} \end{array} \end{array}$$

where ΔG_sol_ accounts for the polar (ΔG_GB_) and non-polar (ΔG_SA_) solvation energies. ΔG_GB_ is the polar contribution to the solvation free energy, described by the Generalized Born (GB) calculation, while ΔG_SA_, the non-polar solvation energy, is calculated from the solvent accessible surface area (SASA). TΔS is the conformational entropy of binding and usually calculated by the normal-mode analysis. The aim of this method is to further understand the effect of each residue on the release process of tacrine, and the main results show that van der Waals, electrostatic interaction, and non-polar solvent effects provide the main driving force for substrate binding, while polar solvent energy plays a negative role.

### RAMD MD Simulations for Detecting Tacrine Release Channels

In order to identify the possible channels for ligand transportation from active site to the outside of the protein, the combined random acceleration molecular dynamics and MD simulations (RAMD MD) (Lüdemann et al., 2000; Vashisth and Abrams, 2008) have been carried out by using NAMD 2.9 software (Phillips et al., 2005). Proteins and ligands were described with ff14SB (Maier et al., 2015; Shao and Zhu, 2018) and GAFF force fields (Wang et al., 2004), respectively. The initial structure of the BChE–tacrine system was derived from the equilibrium state of MM MD simulations. In the RAMD MD approach, a small randomly oriented force is added to the center of the mass of tacrine to identify the possible channels of the ligand release. The direction of the random force is kept for a certain period of time. During this time, if tacrine displacement reaches the threshold parameter, the direction of the force is maintained, otherwise, a new direction will be selected randomly. When the ligand escapes from its initial position, the classic MD simulation will be performed to balance the sampling, which avoided the error arising from the higher random force. In the present work, random accelerations of 0.10, 0.15, 0.20, 0.25, 0.30, 0.35, 0.40, 0.45, and 0.50 kcal/Å/g and thresholds of 0.5, 0.4, 0.3, and 0.2 Å were applied to the initial model, respectively. Totally, 144 RAMD MD trajectories were acquired to identify possible delivery channels of tacrine by statistics analysis.

### Umbrella Sampling

**Figure 4:**
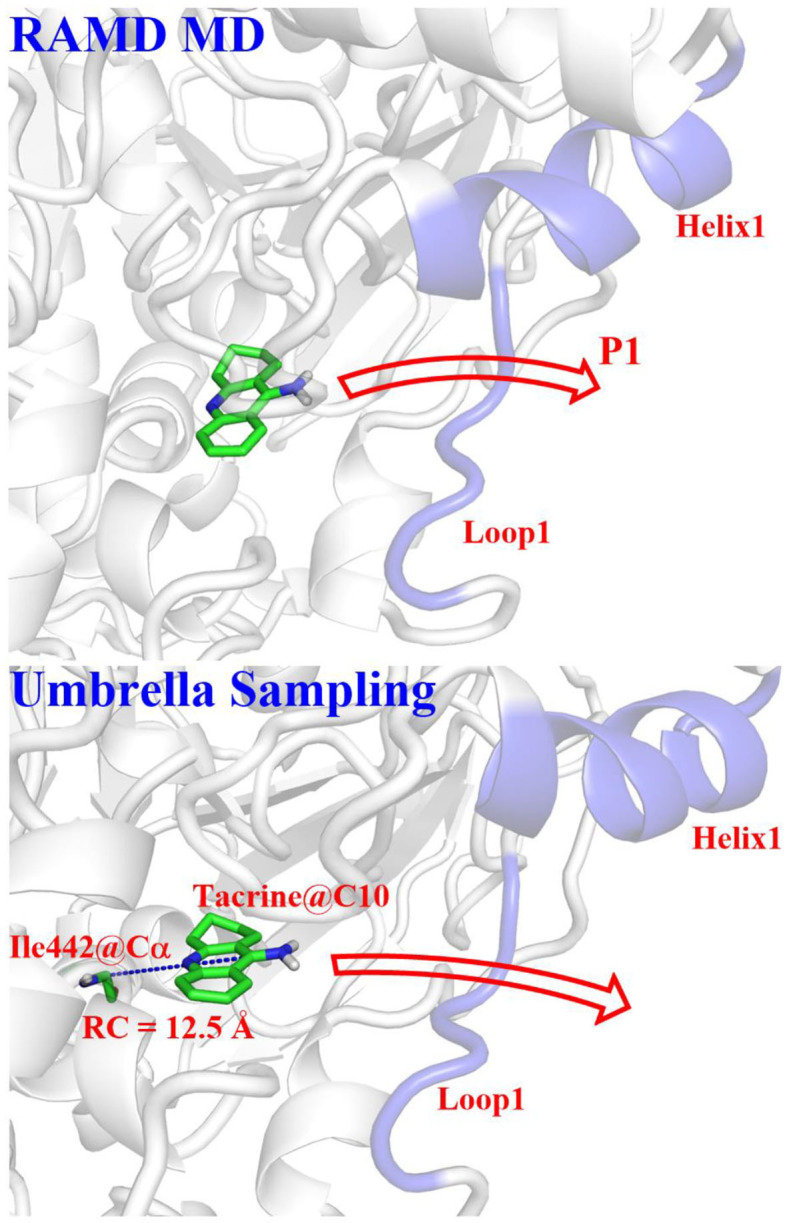
Definitions of reaction coordinate for the tacrine delivery. RC, the distance between the C10 of tacrine and the Cα of Ile442.

Based on the statistics of the RAMD MD simulations, four possible channels were generated. The umbrella sampling (US) technique (Kästner, 2011) was applied to map out the free energy profiles of the most favorable pathway to further identify the thermodynamic and dynamics properties. In this process, the conformational changes of the protein can also be observed. The initial model was constructed based on the stable snapshot structure in the conventional MD simulations. Based on the most probable channel determined from RAMD MD simulations, the distance between the C10 atom of tacrine and the Cα of Ile442 was chosen as the reaction coordinate (RC) for the substrate transportation (see Figure 4), which varies from 12.5 to 32.5 Å with a 0.5-Å interval for two adjacent windows. Afterwards, for each window, 40 ns MM MD simulations with an appropriate biasing harmonic potential were performed along the reaction coordinate to ensure a reasonable overlap for all windows. The last 7 ns trajectory data from the all windows were analyzed by the weighted histogram analysis method (WHAM) to generate the potential of mean force (PMF) (Kumar et al., 1992). Interactions between the ligand and the protein as well as the role of the key residues were investigated.

## Results and Discussions

### Equilibrium Structure Analysis

**Figure 5:**
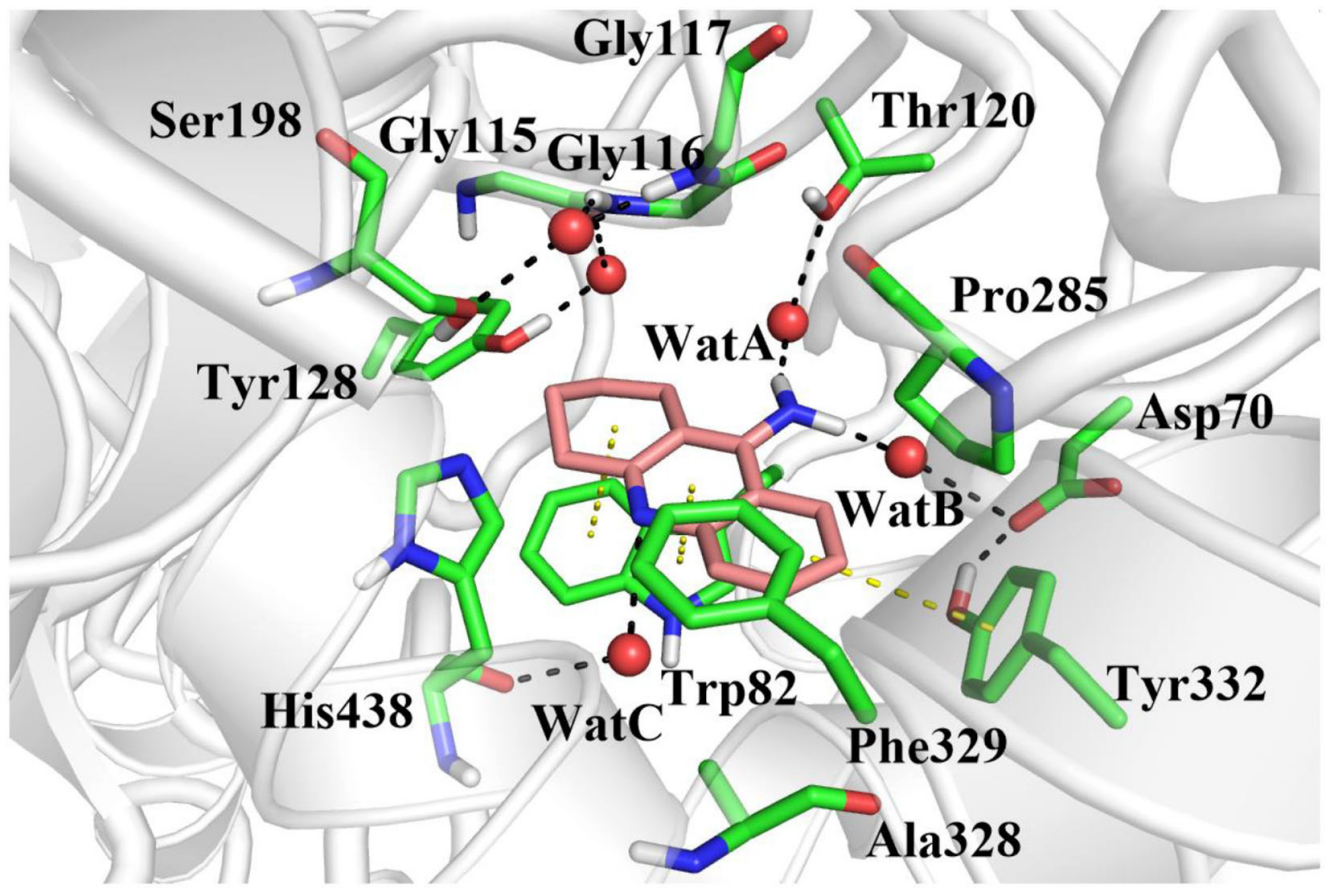
The equilibrium structure of butyrylcholinesterase (BChE)–tacrine complex in the active site after molecular mechanics–molecular dynamics (MM MD) simulations. Key residues are represented as sticks; all oxygen and nitrogen atoms are colored red and blue, respectively. Carbons are colored pink in the ligand and green in residues of the complex. Waters are represented by red spheres, hydrogen bonds are drawn as black dashes, and aromatic stacking are drawn as yellow dashes.

**Figure 6:**
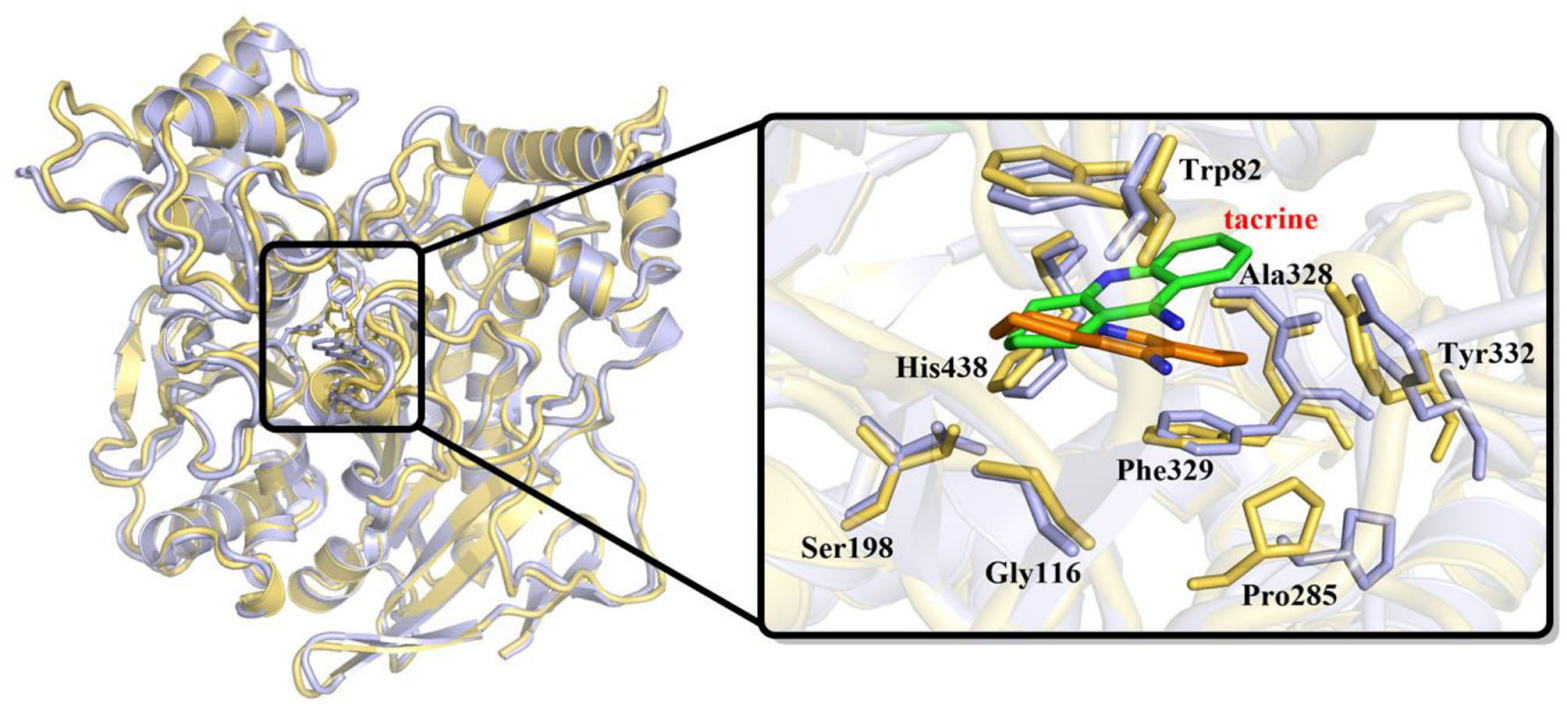
Overlap of initial crystal structure and stable state after molecular mechanics–molecular dynamics (MM MD) simulations. Tacrine in initial crystal structure is represented as green sticks; in MD simulations, structure is represented as orange sticks.

In order to investigate the stability of the enzyme–substrate complex structure for subsequent detection of possible substrate release pathways, long time MM MD simulations have been carried out. On the basis of the root-mean-square deviation (RMSD) results of the backbone atoms, the system approaches equilibrium after 40 ns (see Figure S1), which suggests that the interaction between the ligand and BChE reaches an equilibrium state at that time. Thus, the last 20 ns sampling snapshots have been used for the following analysis. The representative snapshots from the final MD simulation are displayed in Figure 5, which shows that tacrine has been immobilized in the active site of BChE. At that time, strong face-to-face π-π stacking interactions from the aromatic planes of Trp82 have been discovered to stable the substrate in the active site, which plays an essential role in the whole binding model. As shown in Figure 5, it is found that not only a face-to-face π-π stacking interactions from the aromatic planes of Trp82 but also an edge-to-face π-π stacking was formed between the benzene ring from tacrine and Tyr332 immediately. Furthermore, there existed a series of sound van der Waals interaction between the tacrine and the residues of Asp70, Trp82, Gly115, Gly116, Gly117, Thr120, Ala328, Phe329, Tyr332, and His438, which play crucial roles in stabilizing the substrate in the active site. In addition, two localized water molecules (WatA and WatB) in the binding gorge region have been observed to form hydrogen bonds with the amino group of the aromatic ring. Meanwhile, we found that another water molecule (WatC) also plays a crucial role in protein–tacrine association by mediating interactions between the aromatic nitrogen and the main chain carbonyl of His438. Beyond that, a significant number of water molecules have been observed to stay close to the tacrine binding site and form a network of hydrogen bonds to assist the tacrine stabilization. Therefore, the tacrine was stabilized by those abundant hydrogen bonds and strong aromatic stacking aromatic stacking. After 100 ns simulations, the interatomic distances between key residues and tacrine are collected, which is listed in Figure S2, showing good consistence with the X-ray structure. Moreover, the protein and location of key residues in substrate binding, such as Trp82, Gly116, Ser198, Pro285, Ala328, Phe329, Tyr332, and His438, are also extracted to compare with that in the crystal structure by overlap technology. As shown in Figure 6, both protein and active site observed in theoretical calculations and experiment are overlapping well, which means that our calculated model is reasonable.

### Free Energy Decomposition Identifies “Efficient Amino Acids”

**Figure 7:**
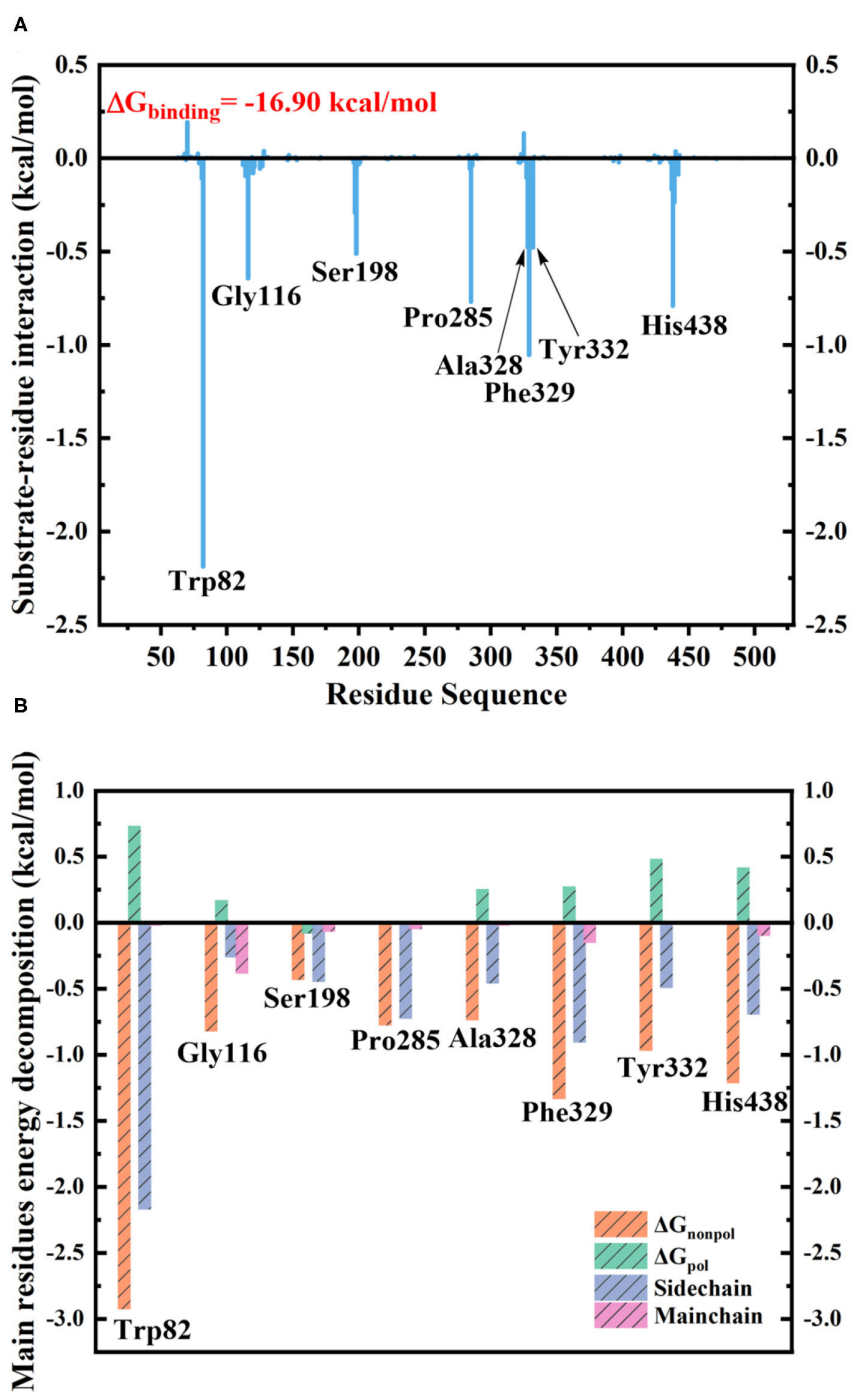
**(A)** Contribution of each residue to butyrylcholinesterase (BChE)–tacrine binding. **(B)** The binding free energy decomposition of the main residues in the BChE–tacrine systems.

**Figure 8:**
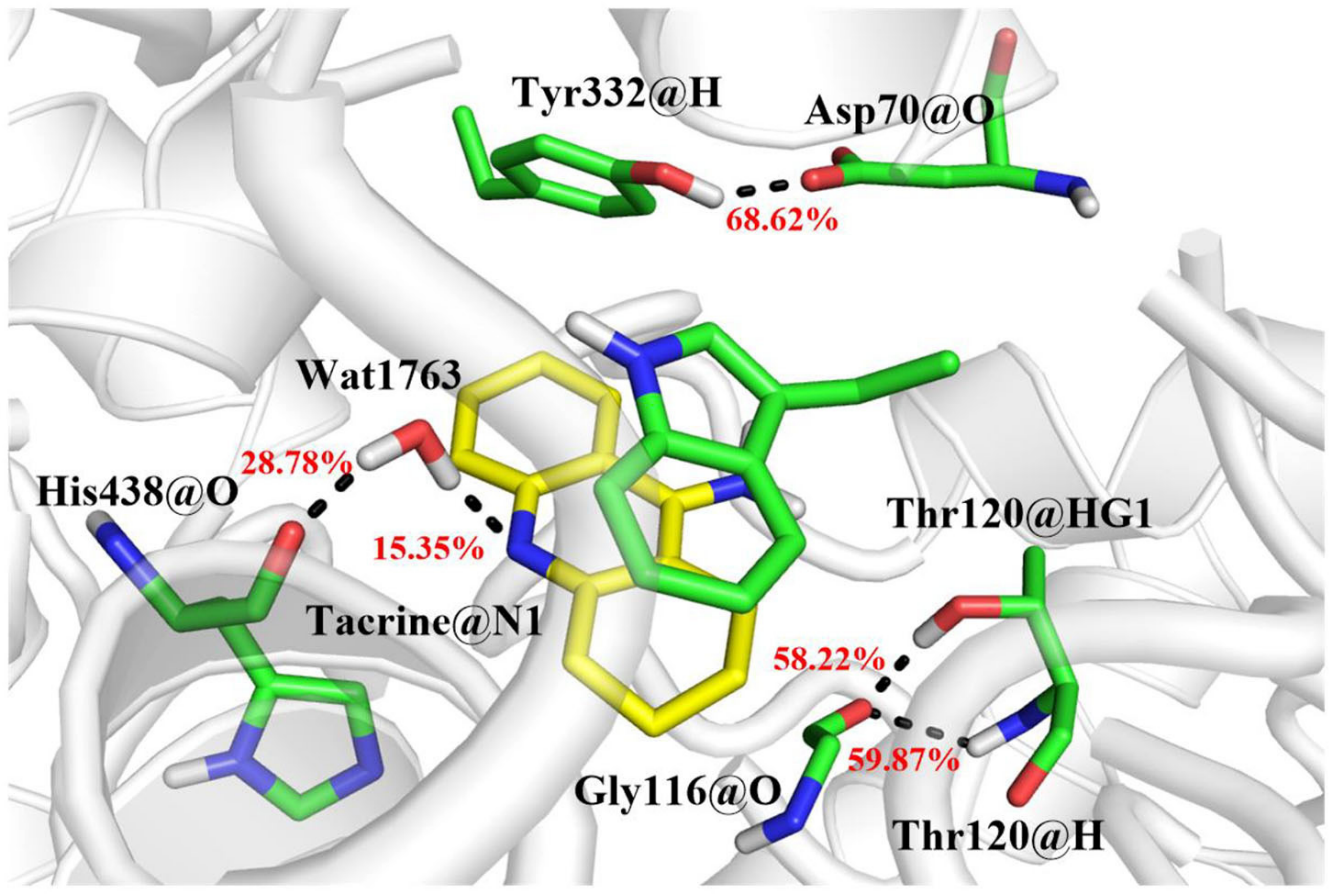
The hydrogen bond network in the active site and the probability of bonding are labeled in red.

The MM/GBSA method has been used to analyze the contribution of all amino acids to the binding free energy of BChE–tacrine complex systematically by employing 500 snapshots from the last 5 ns in MM MD simulations. The total binding free energy of the complex was −16.90 kcal/mol that is constituted by the integrated effects of van der Waals (ΔE_vdW_), electrostatic (ΔE_ele_), non-polar solvation (ΔG_SA_), and polar solvation interaction (ΔG_GB_) (see Table S2), and the ΔE_vdW_ is the most contributor that is benefit for tacrine binding. Moreover, the binding energy contributions of individual amino acids were also calculated, as we can see in Figure 7A, the residues supported interactions with the ligand directly and outstanding contributions to the binding energy have been labeled specifically. We divide van der Waals (ΔE_vdW_), electrostatic (ΔE_ele_), non-polar solvation (ΔG_SA_), and polar solvation interaction (ΔG_GB_) into ΔG_non−pol_ and ΔG_pol_. ΔG_non−pol_ equals to the sum of ΔE_vdw_ and ΔG_SA_, and ΔG_pol_ is comprised of the electrostatic contribution (ΔE_ele_) and polar solvation interaction (ΔG_GB_). Meanwhile, the energy decomposition of these main residues including Trp82, Gly116, Ser198, Pro285, Ala328, Phe329, Tyr332, and His438 has been depicted in Figure 7B. ΔG_non−pol_ of these main residues are −2.93, −0.82, −0.43, −0.78, −0.74, −1.33, −0.97, and −1.22 kcal/mol; ΔG_pol_ of these main residues are 0.74, 0.17, −0.08, 0.01, 0.25, 0.28, 0.49, and 0.42 kcal/mol. As demonstrated in Table 1, for these main residues, van der Waals, electrostatic, and non-polar solvated interactions of BChE–tacrine are favorable in the binding of the complex. Polar interaction is 15.16 kcal/mol, which consists of electrostatic interaction and polar solvation effect, playing an unfavorable role for binding. However, it can be counteracted by non-polar interaction (−32.06 kcal/mol) that is much favorable for the binding, which comprises of van der Waals and non-polar solvation energy. Therefore, the BChE and tacrine can be bound with each other at last considering these above factors. Among them, Trp82, Tyr332, and His438 have a significant contribution to the binding. Trp82 exhibited a particularly high correlation with tacrine especially, which can be justified by the fact that this residue forms parallel strong π-π stacking with tacrine, and the van der Waals interactions and non-polar solvent effects are favorable for the substrate binding. Tacrine forms an edge-to-face π-π stacking with Tyr332. Meanwhile, His438 provides a hydrogen bond with tacrine molecule to stabilize it in the active site. The equilibrium structure shows that Gly115, Gly116, and Gly117 also form a hydrogen bond network around the ligand, which is the main source of their binding energy contribution. At the same time, Asp70 forms a water-mediated hydrogen bond with the ligand. These interactions can be also acquired clearly by snapshots sampling in different windows during the release process of tacrine. In addition, the possibility of hydrogen bonds forming between tacrine and BChE as well as surrounding water molecules is also analyzed in detail by the last 10 ns MD simulation trajectory, as shown in Table 2 and Figure 8. The serial number of atoms in tacrine is listed in Figure S3. The probability of hydrogen bonding between Tyr332 and Asp70 is 68.82%. There is a water-mediated hydrogen bond between tacrine and His438 with the probability of 28.78 and 15.35% for each of them, and Gly116 and Thr120 form a hydrogen bond network around the ligand with the probability of 58.22 and 59.87%, which means that they also make a contribution to stabilize the ligand.

**Table 1:** The mean and standard deviation (M ± SD) of binding free energy decomposition of key residues in butyrylcholinesterase (BChE)–tacrine complexes (kcal/mol).

**Residue**	**ΔE_**vdW**_**	**ΔE_**ele**_**	**ΔG_**GB**_**	**ΔG_**SA**_**	**$\Delta G_{\text{binding}}^{\text{a}}$**
Trp82	−2.54 ± 0.52	−0.39 ± 0.29	1.13 ± 0.45	−0.39 ± 0.09	−2.19 ± 0.41
Gly116	−0.67 ± 0.16	−0.27 ± 0.09	0.44 ± 0.09	−0.15 ± 0.02	−0.65 ± 0.19
Ser198	−0.40 ± 0.10	−0.07 ± 0.06	−0.01 ± 0.11	−0.03 ± 0.01	−0.51 ± 0.17
Pro285	−0.66 ± 0.17	−0.05 ± 0.12	0.06 ± 0.09	−0.12 ± 0.03	−0.77 ± 0.19
Ala328	−0.64 ± 0.18	−0.27 ± 0.14	0.52 ± 0.20	−0.10 ± 0.02	−0.48 ± 0.23
Phe329	−1.24 ± 0.28	−0.28 ± 0.12	0.56 ± 0.11	−0.09 ± 0.02	−1.06 ± 0.24
Tyr332	−0.83 ± 0.30	−0.03 ± 0.18	0.52 ± 0.20	−0.14 ± 0.02	−0.48 ± 0.28
His438	−1.06 ± 0.26	0.16 ± 0.29	0.26 ± 0.22	−0.16 ± 0.02	−0.79 ± 0.24

*${{\Delta G}_{binding}}^{a}={\Delta E}_{vdW}+{\Delta E}_{ele}+{\Delta G}_{GB}+{\Delta G}_{SA}\;$*.

**Table 2:** Hydrogen bonds formed between tacrine and butyrylcholinesterase (BChE) and the corresponding percent analysis with the last 10 ns trajectories in the MD simulations.

**Donor**	**Acceptor H**	**Acceptor**	**Percent (%)**
Asp70@O	Tyr332@H	Tyr332@O	68.62
Gly116@O	Thr120@H	Thr120@N	59.87
Gly116@O	Thr120@HG1	Thr120@OG1	58.22
His438@O	Wat1763@H2	Wat1763@O	15.89
His438@O	Wat1763@H1	Wat4805@O	12.89
Tacrine@N2	Wat1763@H1	Wat4805@O	8.55
Tacrine@N2	Wat1763@H2	Wat4805@O	6.80

**Figure 9:**
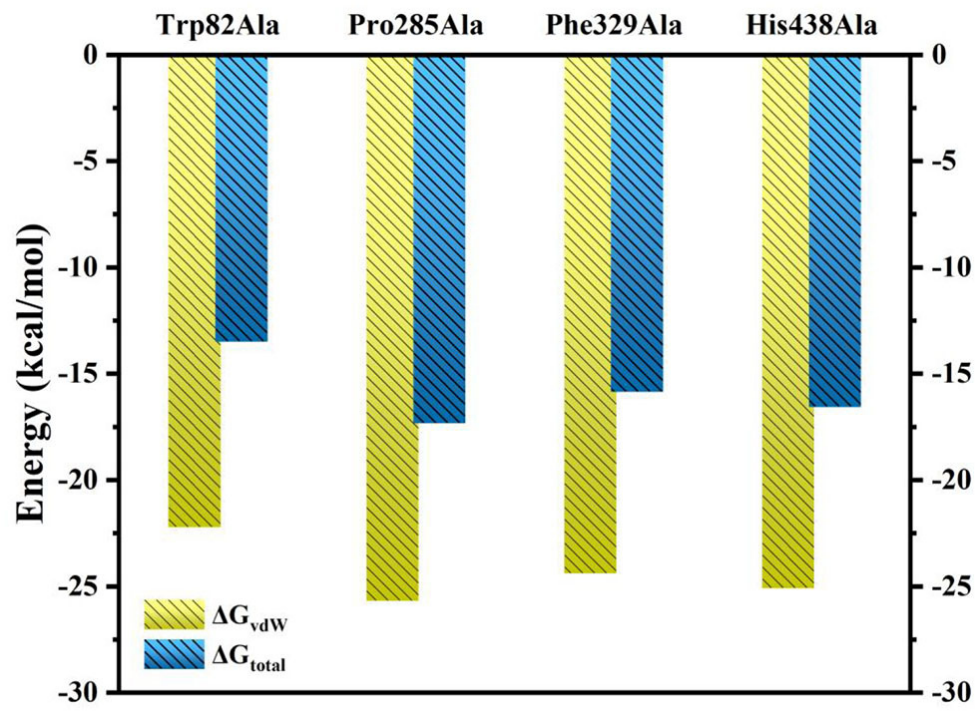
Comparison of ΔG_vdW_ and ΔG_total_ for Ser79Ala, Trp82Ala, Thr120Ala, Tyr332Ala, and His438Ala.

Furthermore, to identify the role of the specific residues, four key residues of Trp82, Pro285, Phe329, and His438 are selected to take site-directed mutations of alanine, since they contributed the most to the entire substrate and protein binding process according to the MM/GBSA calculation results. A single virtual alanine scanning calculations was made for each of them by 100 snapshots from last 5 in 100 ns MM MD simulations for wild system to verify their contribution to protein–ligand complex binding. These mutant systems are named as Trp82Ala, Pro285Ala, Phe329Ala, and His438Ala, respectively. Detailed results are summarized in Table 3 and Table S3. More importantly, if we carefully analyze all data presented there, we could find that the van der Waals interactions between ligand and protein dominate most to the binding free energy. To illuminate this, the comparison between calculated G_vdW_ and G_total_ is plotted in Figure 9. Table 3 described the difference value of binding free energy between the wide and mutation type. Here, Trp82 is the biggest contributor among the four residues involved in the binding of ligand through the strong π-π stacking. Compared to the wild type, the energies of Trp82Ala decrease to −13.48 kcal/mol, 4.43 kcal/mol less than the wild type. For Phe329, coming in the second, the energies decrease to −17.32 kcal/mol. His438 decreases the binding energy of 2.30 kcal/mol after mutating to Ala residue. It can be found that there is an aromatic stacking with Trp82 and a weak hydrogen bonding between the N7 atom of tacrine and His438 (3.2 Å) experimentally (Nachon et al., 2013). Therefore, our calculated results are in good agreement with the experimental data, suggesting that residues Trp82 and His438 contribute significantly to binding of tacrine. The mutation of these residue leads to a significant decrease in binding affinity. In addition, the mutations of Pro285Ala also cause a tiny reduction in binding energy. It is probably due to the reason that this residue does not interact directly with ligands and would be neglected by the calculation error. Moreover, there still exist a weak interaction between the mutant alanine and the ligand. If one residue was changed, the interactions around tacrine may be changed to some extent.

**Table 3:** The mean and standard deviation (M ± SD) of binding free energy of wild and mutant systems (kcal/mol).

**Mutated system**	**Wild**			**Mutant**			**ΔG*_***wild***_* – ΔG*_***mutant***_***
	**ΔG*_***gas***_***	**ΔG*_***solv***_***	**ΔG*_***binding***_* – *_***wild***_***	**ΔG*_***gas***_***	**ΔG*_***solv***_***	**ΔG*_***binding***_* – *_***mutant***_***	
Trp82Ala	−32.02 ± 2.41	14.11 ± 1.73	−17.91 ± 1.70	−27.17 ± 1.81	13.69 ± 1.42	−13.48 ± 1.37	−4.43 ± 0.85
Pro285Ala	−32.02 ± 2.41	14.11 ± 1.73	−17.91 ± 1.70	−31.44 ± 2.39	14.12 ± 1.74	−17.32 ± 1.67	−0.59 ± 0.33
Phe329Ala	−32.02 ± 2.41	14.11 ± 1.73	−17.91 ± 1.70	−29.81 ± 2.43	13.96 ± 1.68	−15.85 ± 1.73	−2.07 ± 0.41
His438Ala	−32.02 ± 2.41	14.11 ± 1.73	−17.91 ± 1.70	−30.41 ± 2.30	13.85 ± 1.68	−16.55 ± 1.63	−1.36 ± 0.51

### Transport Mechanism of Tacrine Delivery

**Figure 10:**
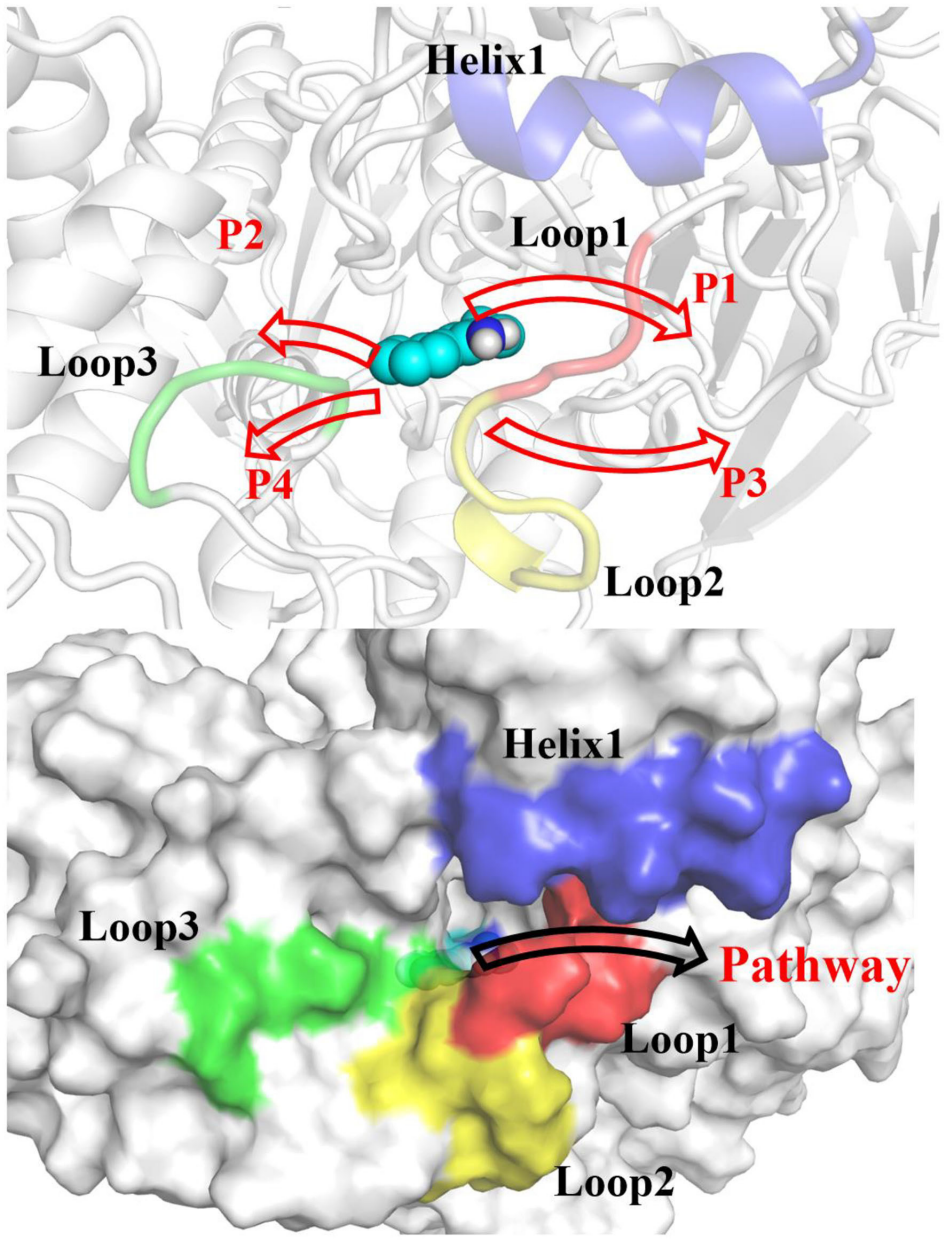
Definition of the possible pathways P1, P2, P3, and P4 for the substrate release based on the random acceleration molecular dynamics molecular dynamics (RAMD MD) simulations.

According to RAMD MD simulations, 144 trajectories were obtained, and four possible pathways were identified, named as P1 (among helix 1 and loop 1), P2 (between helix 1 and loop 3), P3 (through loops 1 and 2), and P4 (between loops 2 and 3) in Figure 10 and Table 4. The possibilities of these channels are 61.11, 29.86, 6.25, and 2.78% for P1, P2, P3, and P4, respectively. As we can see from Figure 10, there is a cavity in the P1 direction, which will facilitate the release of the ligand. Therefore, as we can see in Table 4, path P1, located between helix 1 and loop 1, is recognized as the predominant pathway for the release of substrate, which will be discussed in detail.

**Table 4:** The location, number, and possibility of trajectories for tacrine delivery pathways based on random acceleration molecular dynamics molecular dynamics (RAMD MD) simulations.

**Pathway**	**Location**	**Number**	**Possibility**
P1	Helix 1, loop 1	88	61.11%
P2	Helix 1, loop 3	43	29.86%
P3	Loop 1, loop 2	9	6.25%
P4	Loop 2, loop 3	4	2.78%

*The possibility was calculated by the ratio of N_x_/N_total_, where N_x_ is the number of trajectories of pathway P_x_ (x = 1–4), and N_total_ is the number of total trajectories*.

**Figure 11:**
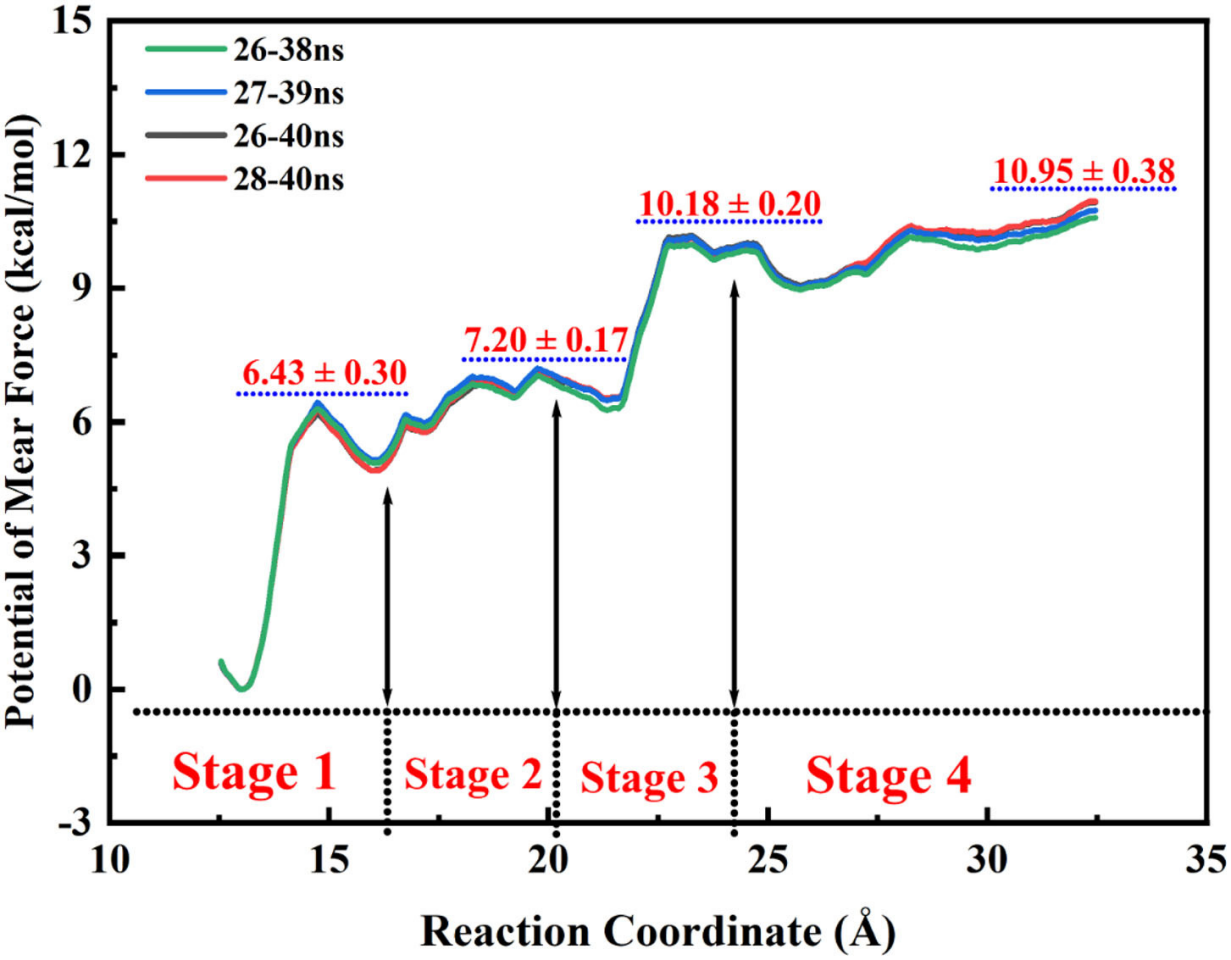
Free energy profiles for the release of tacrine along the reaction coordinate.

**Figure 12:**
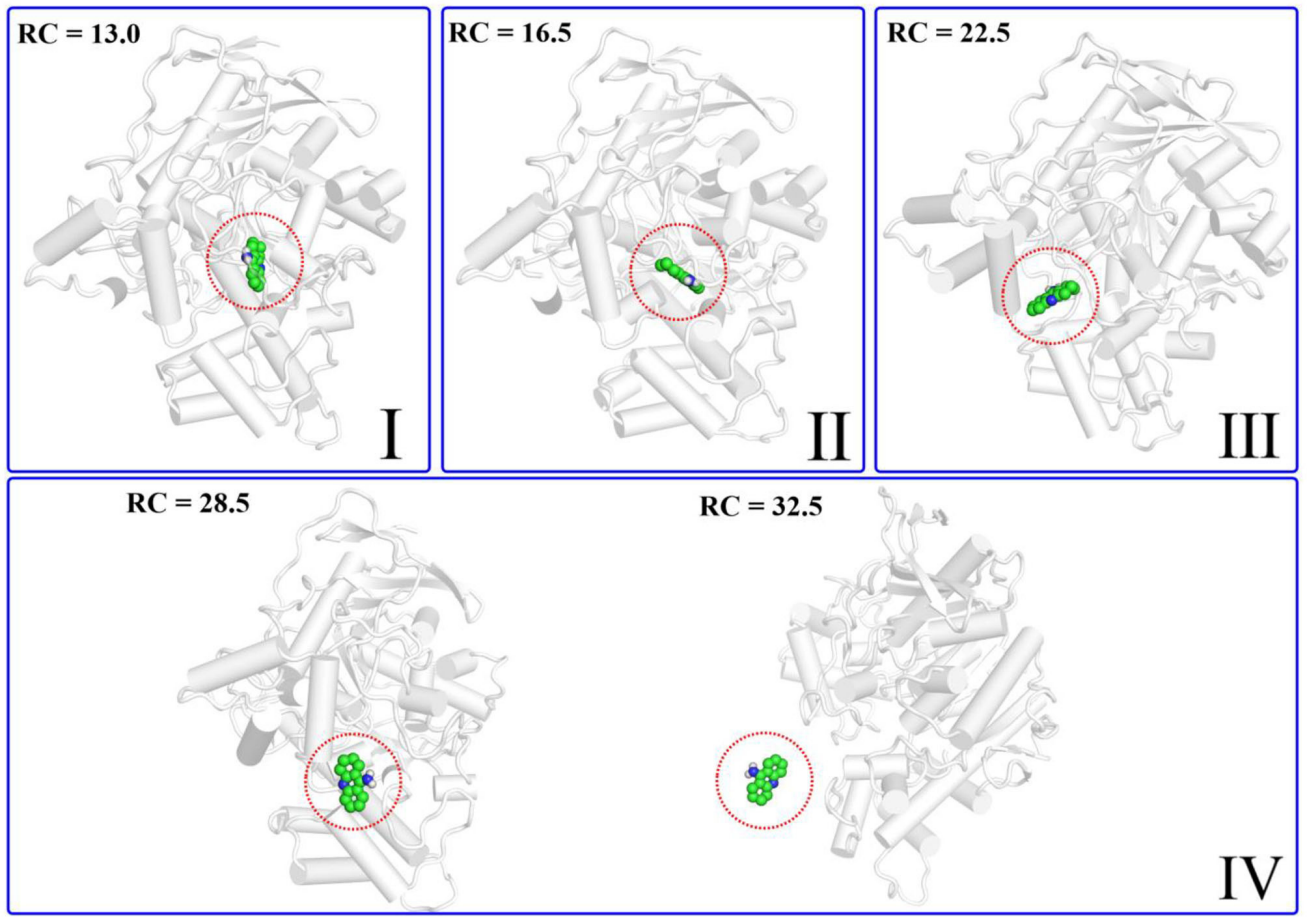
Structure of the protein changes along P1 in the four phases of the tacrine release determined by umbrella sampling simulations.

**Figure 13:**
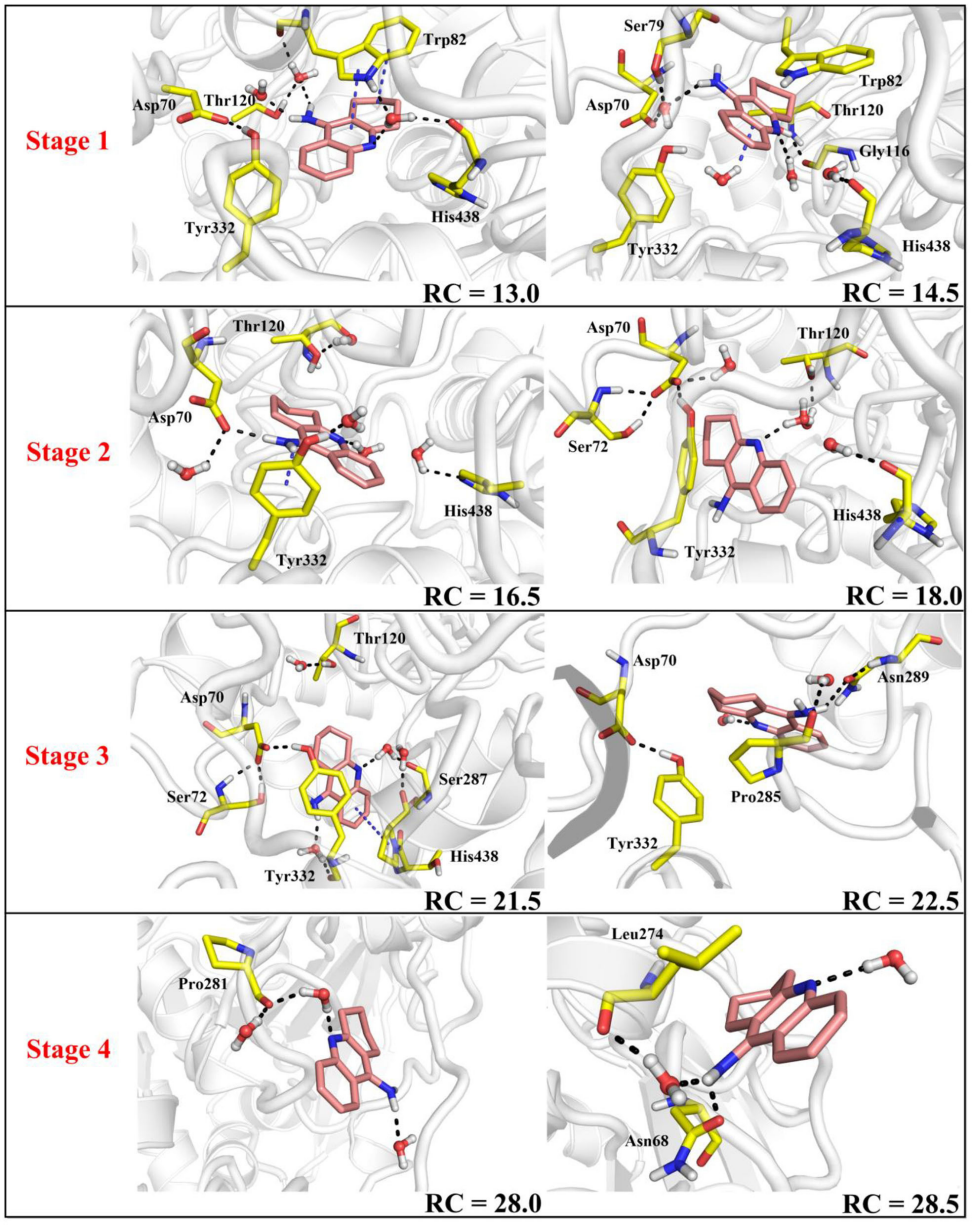
Key residues and their conformations in the active site from different windows along the reaction coordinate. Key residues and waters are represented as sticks. Hydrogen bonds are drawn as black dashes; aromatic stacking are drawn as blue dashes.

According to the escaping direction of tacrine for the most favorable channel P1, the distance between the C10 atom of tacrine and the Cα atom of Ile442 was selected as the reaction coordinate (RC) (see Figure 4). In order to obtain a more reliable substrate release mechanism and visualized potential of the mean force (PMF), a total of 1,640 ns MD simulations combined with the umbrella sampling technique along the reaction coordinate from 12.5 to 32.5 Å were carried, and 41 windows were collected. For each window, a series of biasing harmonic potential of 30 kcal/mol was added to ensure full sampling and enough overlapping between neighboring windows (Figure S4). Figure S5 shows the fluctuations of the backbone of BChE, indicating that the trajectories reach a steady state after 20 ns. Moreover, in order to verify the convergence of PMF curves (Magistrato et al., 2017), different time periods and districts are considered (see Figures S6, S7), different time periods and districts are considered (see Figures S6, S7), which implies that the free energy profile probably reach convergence after 25 ns. Therefore, the energy profiles of different sampling time durations of 26–40, 28–40, 27–39, and 26–38 ns have been compared in Figure 11. Clearly, the relative free energy profiles for different sampling time durations are essentially the same with the maximal SD of 0.38 kcal/mol, showing reliable convergence of MD simulations for the PMF calculations. Based on the free energy and the conformational changes of residues and proteins shown in Figures 11–13, the escape process of substrates from the proteins is divided into four consecutive stages.

**Figure 14:**
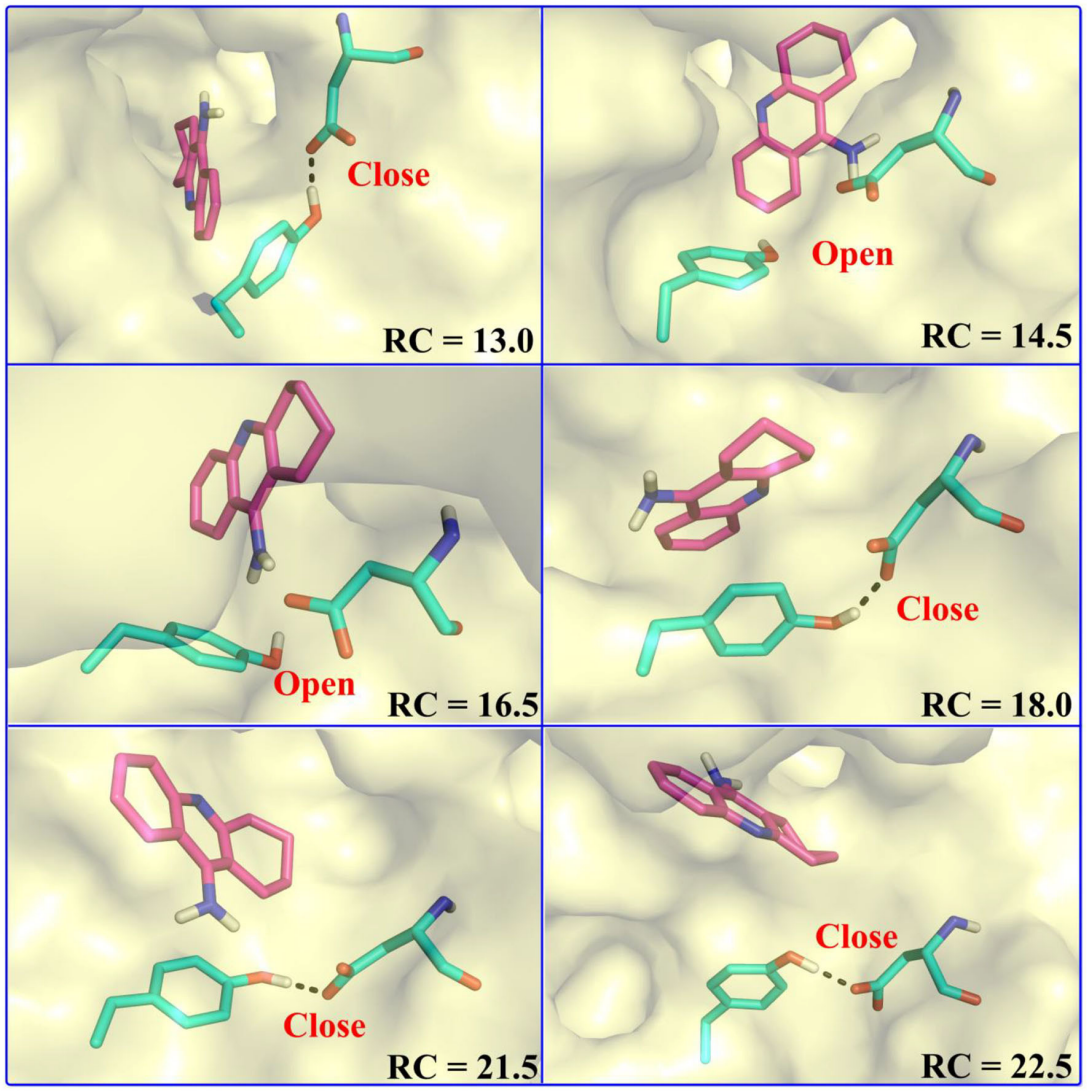
Representative states of the doorkeeper residues along the substrate release process.

In the first stage (12.5 Å ≤ RC < 16.0 Å), the delivery channel is totally closed (shown in Figure 14). At 13.0 Å, Tyr332 forms a hydrogen bond with Asp70 to assist the “gate” totally close, and a stable face-to-face π-π stacking formed between tacrine and Trp82 indole ring in the active site. At the same time, the amino group of tacrine forms two water-mediated hydrogen bonds with the water. In addition, van der Waals interactions can be detected from the main chains of Thr120 and Gly115. Both hydrogen bond and van der Waals interactions provide the necessary force to stabilize tacrine at this stage. Then, the hydrogen bond between Tyr332 and Asp70 is broken, causing the “gate” beginning to open; the side chain of Asp70, one of the door keepers, provides a driving force through a new water-mediated hydrogen bond with the substrate at 14.5 Å of reaction coordinate, resulting in the tacrine beginning to move to the “door” as seen in Figure 13. In the second stage (16.0 Å ≤ RC < 20.0 Å), with the reaction coordinate changing, the substrate tries to break away from the active site, leading the water-mediated hydrogen bond between tacrine and Asp70 broken as shown in RC = 16.5 Å and forming a new aromatic hydrogen bond between the amino and Tyr332. We can observe that Tyr332, as another door keeper, turns on one side gradually as a “swinging gate.” At this stage, these two door keepers (Asp70 and Tyr332) interact with the substrate successively and get the hydrogen bond between Tyr332 and Asp70 broken, causing the “gate” beginning to open. At the same time, a new water-mediated hydrogen bond is formed between the carbonyl of Phe329 and substrate, which also provides a driving force for tacrine delivery. Then, water provides a driving force for the substrate escape through a new hydrogen bond. Meanwhile, the “swinging gate” gets back to the original “close” state; the substrate moves toward the protein “cavity” where the number of residues around tacrine is obviously reduced. In the third stage (20.0 Å ≤ RC < 23.5 Å), the newborn hydrogen bond with Tyr332 is broken, following the formation of a water-mediated hydrogen bond with Tyr332 for a short time, and the aromatic nitrogen atom of the tacrine forms a water-mediated hydrogen bond with Ser287. Meanwhile, the main chain of Pro285 forms two hydrogen bonds with tacrine. Then, the conjugate ring flips and tries to move toward outside. The amino group of tacrine forms a strong hydrogen bond with Asn289; the aromatic nitrogen atom of the tacrine forms a hydrogen bond with the water. This flipping resulted to the fluctuation in the third stage in the free energy profiles along the reaction coordinate. At this stage, tacrine tries to escape from the active site; Asn289, closer to the entrance of the pocket, provides a sound driving force by a hydrogen bond. Especially, Asn289 forms a hydrogen bond with tacrine directly. The specific protein environment makes it difficult for the substrate opening the “swinging gate” and escaping from the pocket facilely. Thus, the detour-forward strategy was adopted for the substrate to release. In the last stage (RC ≥ 23.5 Å), tacrine gets rid of the constraint of the hydrogen bonding with Asn289 and gradually flees away from the constraint of BChE. The interactions around tacrine are obviously reduced and weaken, and thus, the tacrine fluctuates stronger, which caused its conformation changes apparently. The inhibitor looks like traveling a long distance in small difference between two adjacent windows. For example, at 28.0 Å, a water bridge has been identified between Pro281 and N7 of tacrine, and at 28.5 Å, a direct hydrogen bond has been found between Asn68 and –NH_2_ of tacrine. After 32.5 Å, the tacrine escapes from BChE completely, which floats freely that totally exposed to the water environment.

To give a clearer indication of the release process of tacrine and the opening and closing process of the “swinging gate,” we made the illustration of the “gate” during the substrate release, as shown in Figure 14. In addition, to make the release more intuitive, only the key residues, Asp70 and Tyr332, acted as gatekeepers as shown in Figure 14. The conceptual graphs revealing the release process of tacrine are shown Figure 12. As we can see from Figure 14, in the first stage (12.5 Å ≤ RC < 16.0 Å), Tyr332 forms a stable hydrogen bond with Asp70, and the “swinging gate” keeps the tight closing state. The substrate begins to move outward, trying to break away from the active site. Tacrine approaches the “swinging gate” guided by the doorkeeper Asp70, causing the hydrogen bond between two doorkeepers (Asp70 and Tyr332) broken. In the second stage (16.0 Å ≤ RC < 20.0 Å), the “swinging gate” totally open, although that is does not keep a long time. The substrate moves out of the protein under the guidance of doorkeeper Asp70. Then, the “door” gets back to the “close” state. In the last two stages (RC ≥ 20.0 Å), tacrine has reached the cavity outside the “door.” The “door” no longer constrains the release of tacrine, and the “swinging gate” rotates back to the original position.

As shown in Figure 11, this release process is exothermic, while there is a need to overcome an energy barrier of 10.95 kcal/mol. First, the barrier keeps arising from the 13.0 Å of the reaction coordination until almost close to the top at 23.0 Å. The previous three stages are a series of energy increasing steps over constantly. In the first stage, tacrine began to move toward the exit and given rise to the π-π stacking with Trp82 and the hydrogen bond with His438 broken simultaneously. Thus, this is probably the main cause of the barrier when the reaction coordination changes from the 13.0 to 15.0 Å. However, the broken hydrogen bond interaction with Asp70 and Tyr332 may be the primary reason of the risen energy at the second stage. When the reaction coordination moves to the third stage, the free energy fluctuates from 6.2 to 10.1 kcal/mol and reaches the peak of the profiles. It probably comes from tacrine flipping; the flipping leads to the breaking of all non-bond interactions. As to the last stage, tacrine gets rid of the constraint of the hydrogen bonding with Asn289 and Asn68. Thus, there generated an unimpeded path for the substrate delivery, which is probably the main reason of the fluctuation in the free energy profile in Figure 11. Generally, according to the different stages in the delivery mechanism and their thermodynamic properties, the tacrine escaping from BChE will need to overcome a barrier of ~1,0.95 kcal/mol, and π-π stacking interactions with Trp82 are the major factors that affect the overall ligand transportation process. Besides, the hydrogen bond interactions with the residues around the ligand surely affect the transportation process as well.

## Conclusion

In this work, the binding characteristics of BChE and antagonist tacrine complex as well as the release mechanism of ligand have been studied by using extensive MM MD simulations combined with several methods and technologies such as MM/GBSA, RAMD, umbrella sampling, etc., and some significant findings were obtained. First, Trp82, Tyr332, His438, and water bridges have been observed to play a crucial role in substrate binding by hydrogen bonds and π-π stacking interactions, which can be also verified by mutate calculations of key residues. Moreover, the non-polar interaction and side chain effects are discovered as the main contributing factors for tacrine and BChE binding. Second, four possible channels have been identified, and the most favorable channel P1 that located around helix 1 and loop 1 has been chosen to describe the mechanism and thermodynamic properties of substrate delivery process. The barrier for the tacrine release is measured to be around 10.57–10.95 kcal/mol, which mainly comes from the flip of the substrate as well as the broken π-π stacking interactions between Trp82 and tacrine.

Moreover, the hydrogen bond formed between Tyr332 and tacrine also contributes to the barrier. Besides that, the substrate release is a hydrogen-bond-dependent process, especially for the hydrogen bond interactions coming from Tyr332 and Asp70, which are the main factors that influence the delivery of tacrine. It is an intricate and flexural process for the substrate to escape from the deep pocket; thus, a detour-forward strategy was adopted to cross the “swinging gate” and release. Lastly, the key residues Tyr332 and Asp70 forms a “swinging gate,” which shows a dynamical switch mechanism, probably dominating the transportation of tacrine. In summary, the results of this work elucidate the antagonist-binding mechanism in the pocket of BChE on the basis of tacrine and supply detailed information about its dissociation pathway, mechanism, and thermodynamic properties at the atomic level. Nowadays, many derivatives are designed based on tacrine, which have good performance in inhibitor activity (Santos et al., 2016; Jerabek et al., 2017; Okten et al., 2019). Therefore, we hope that this work could provide more dynamic information to design novel BChE inhibitors with better kinetic properties by improving the drug residence time, which will be useful for carrying out rational clinical drug treatment for AD.

## Data Availability Statement

The raw data supporting the conclusions of this article will be made available by the authors, without undue reservation.

## Author Contributions

ZZ and FF performed calculations. ZZ, FF, and YZ analyzed the data. WL, YZ, and CW designed the research. ZZ, FF, YZ, and CW wrote the paper. All authors have approved the final version of the manuscript.

## Conflict of Interest

The authors declare that the research was conducted in the absence of any commercial or financial relationships that could be construed as a potential conflict of interest.
