# Supplementary material for: Molecular Dynamics Revealing a Detour-Forward Release Mechanism of Tacrine: Implication for the Specific Binding Characteristics in Butyrylcholinesterase
Source: Front Chem. 2020 Aug 25;8:730. doi: 10.3389/fchem.2020.00730 (PMC7477934; doi:10.3389/fchem.2020.00730)
Supplement: Supplementary file 1 [file Presentation_1.pdf]

Supporting Information

Molecular Dynamics Studies Revealing a Detour-forward Release Mechanism of Tacrine: Implication for the Specific Binding Characteristics in Butyrylcholinesterase (BChE)

Zhiyang Zhang[1a], Fangfang Fan[2a], Wen Luo[1], Yuan Zhao[1*], Chaojie Wang[1*]

[1]The Key Laboratory of Natural Medicine and Immuno-Engineering, Henan University, Kaifeng, China

[2]School of Biological and Chemical Engineering, Zhejiang University of Science and Technology, Hangzhou, China

[a]Zhiyang Zhang and Fangfang Fan contributed equally to this work.

*** Correspondence:**

Corresponding Author

zhaoyuan@henu.edu.cn (Y. Zhao) wcjsxq@henu.edu.cn (C. Wang)

Contents

Figure S1. 1

Figure S2. 2

Figure S3. 3

Figure S4. 4

Figure S5. 5

Figure S6. 6

Figure S7. 7

Table S1 8

Table S2. 9

Table S3. 10

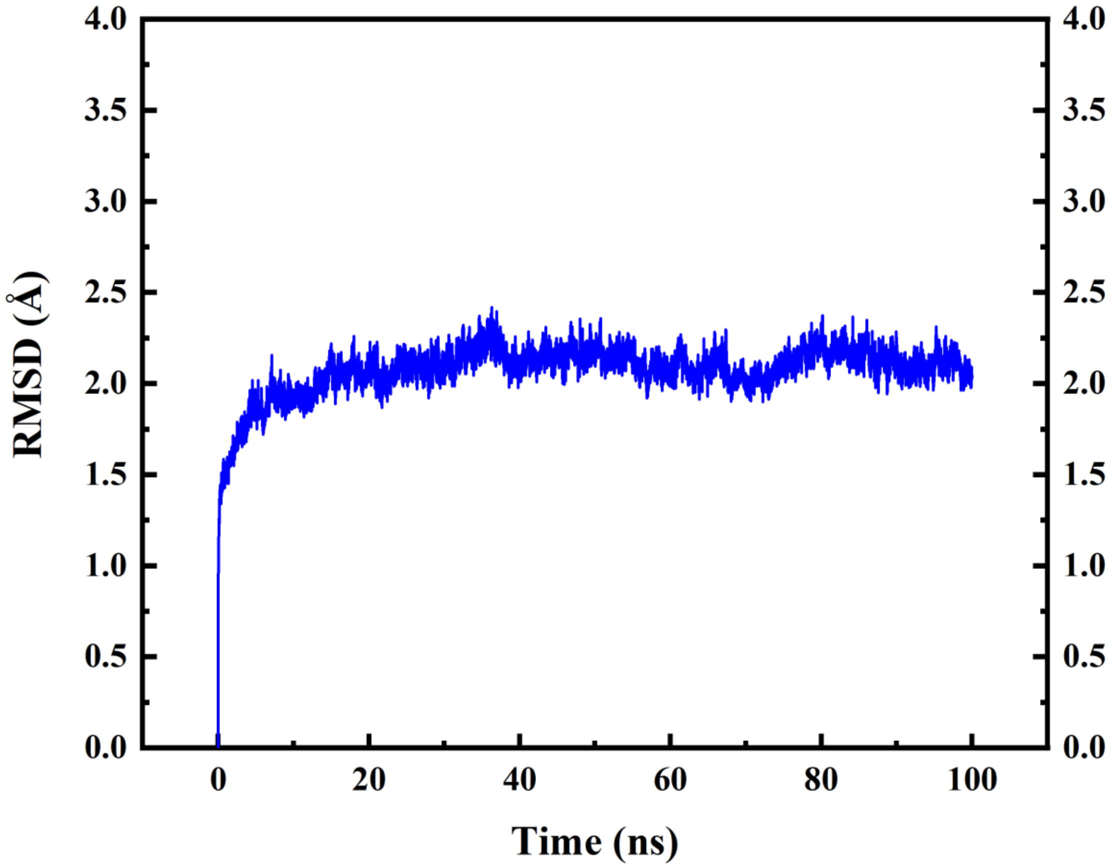

Figure S1. The RMSD of tacrine-BChE complex in MM MD simulations.

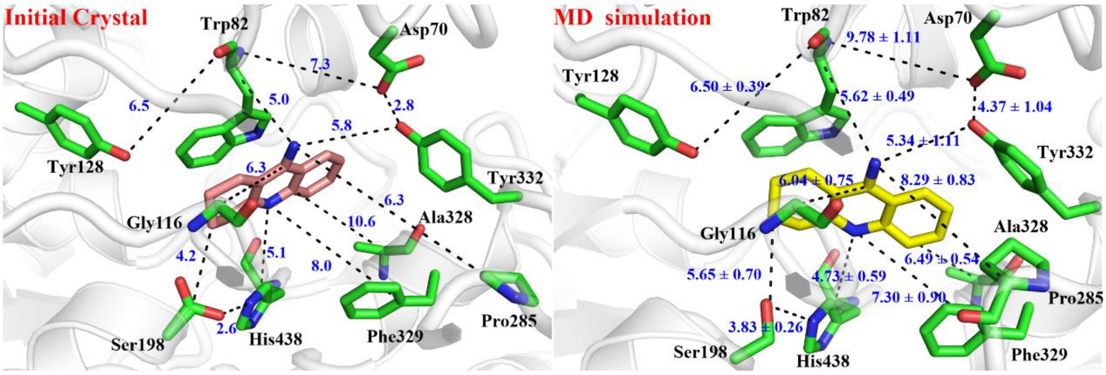

Figure S2. Comparison of X-ray structure residue distance and MD simulated residue distance.

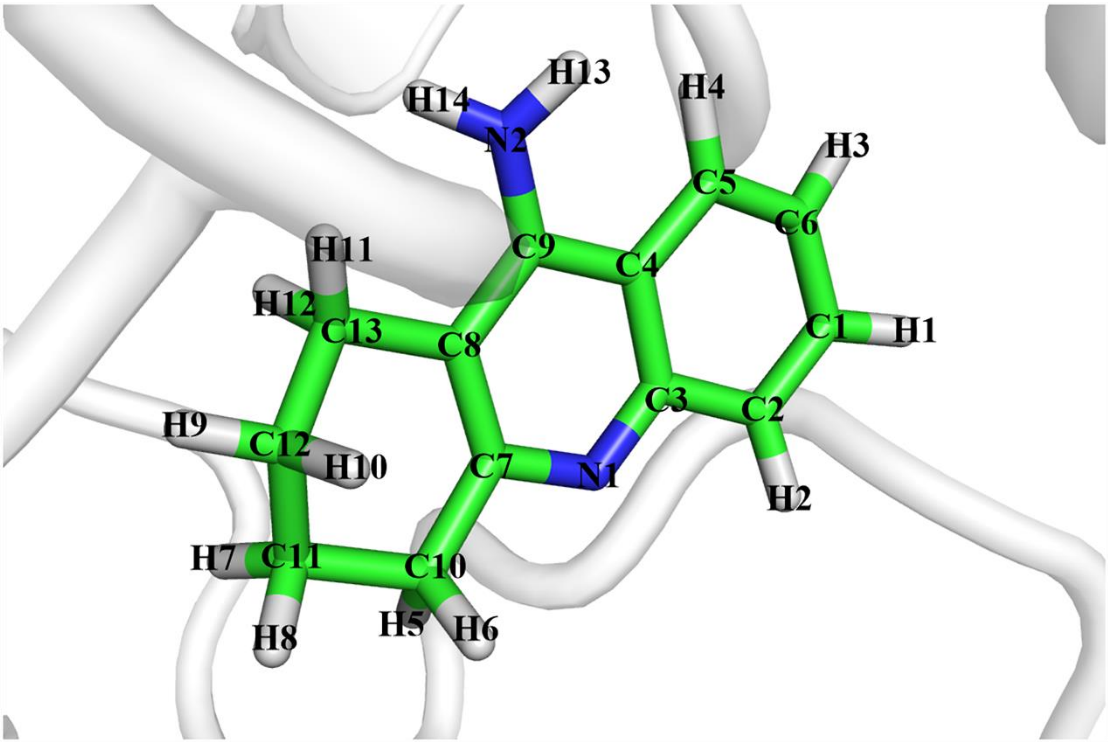

Figure S3. The details and atomic name numbers of tacrine.

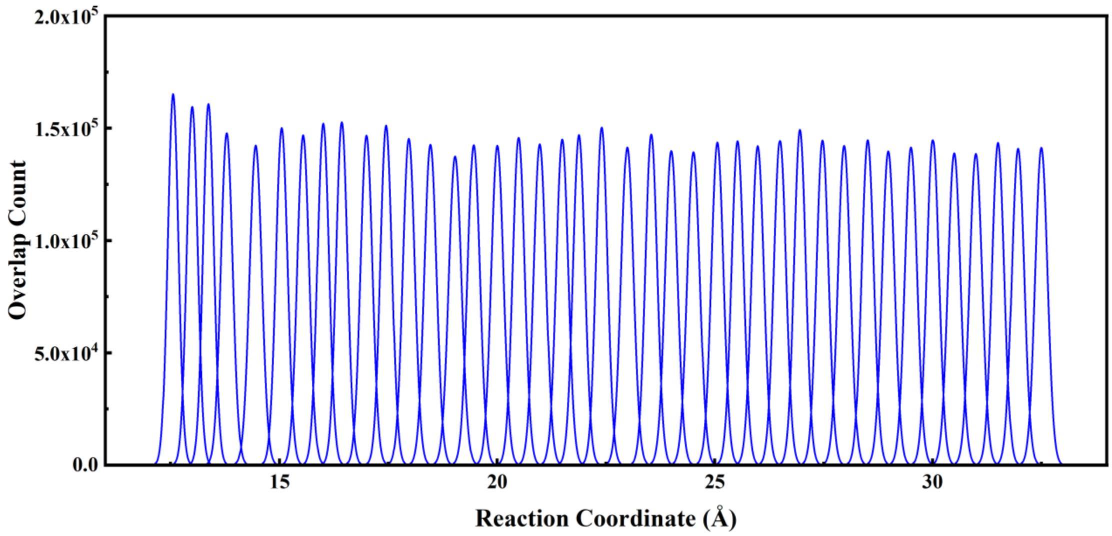

Figure S4. The overlap of windows along P1 pathway based on umbrella sampling. technique.

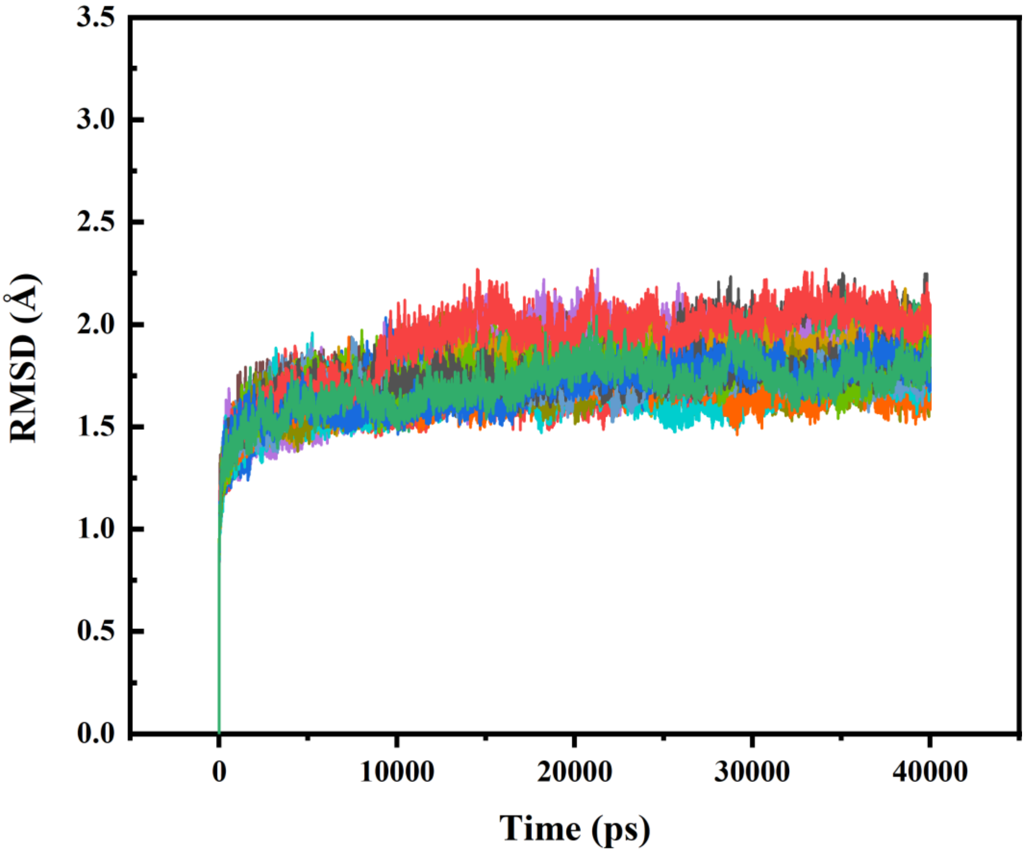

Figure S5. The root-mean-square deviation (RMSD) of all the backbone atoms by total 1640 ns classical MD simulations combined with umbrella sampling technique along P1 pathway.

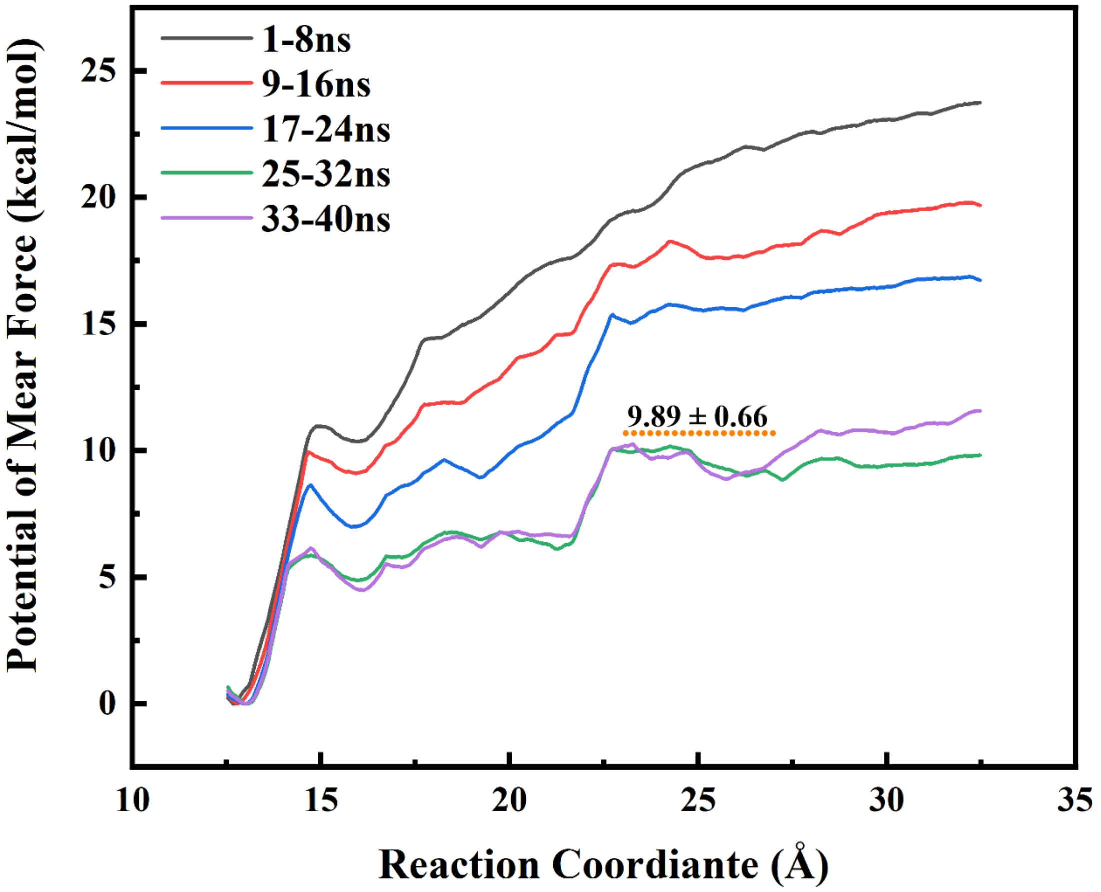

Figure S6. Free energy profiles for the release of tacrine along the reaction coordinate.

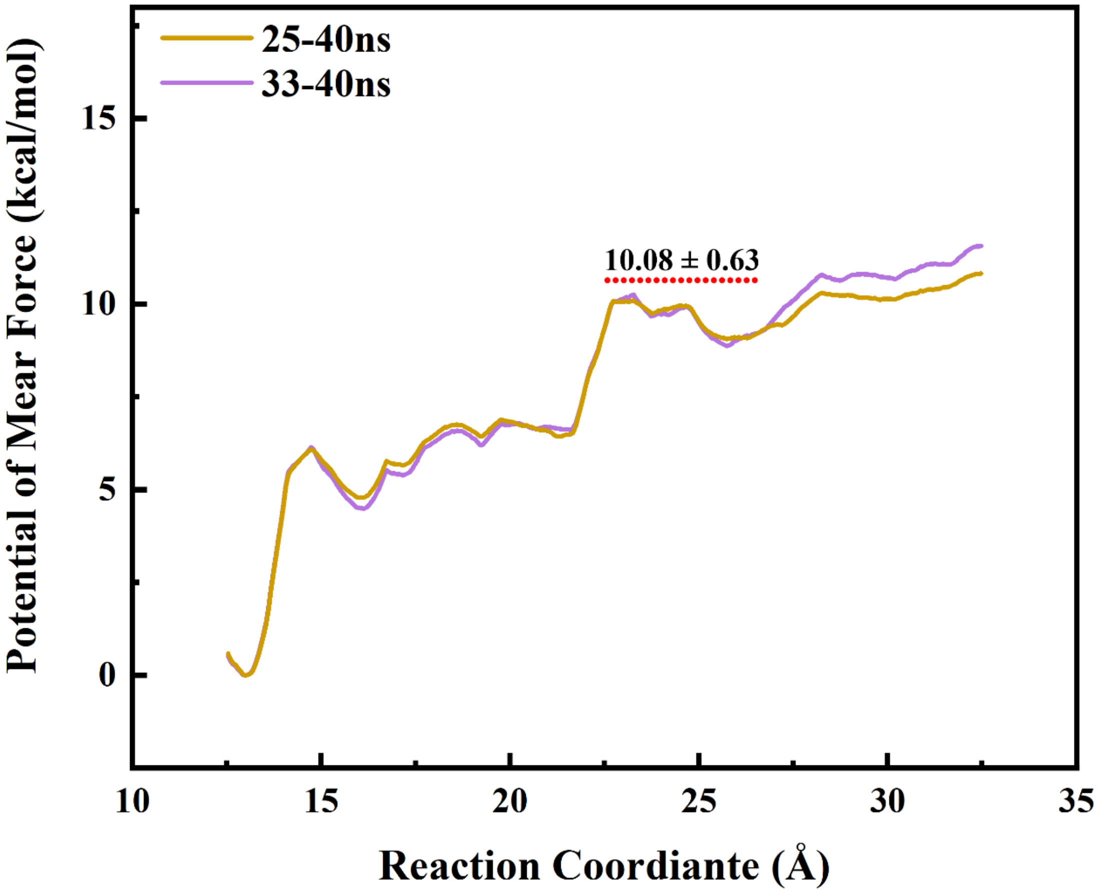

Figure S7. Free energy profiles for the release of tacrine along the reaction coordinate.

Table S1 Comparison of X-ray structure residue distance and MD simulated residue distance.

Reference atom	Reference atom	Distance (Å)	Standard Deviation	Initial Crystal (Å)
**Typ82@Cα**	**Tacrine@N1**	5.62	0.49	5.00
**Gly116@Cα**	**Tacrine@C1**	6.04	0.75	6.30
**Ser198@Cβ**	**Tacrine@C5**	5.65	0.70	4.20
**Pro285@Cα**	**Tacrine@N1**	8.29	0.83	10.60
**Ala328@Cα**	**Tacrine@C8**	6.49	0.54	6.30
**Phe329@Cγ**	**Tacrine@N2**	7.30	0.90	8.00
**Tyr332@Cα**	**Tacrine@N1**	5.34	1.11	5.80
**His438@Cα**	**Tacrine@N2**	4.73	0.59	5.10
**Trp82@O**	**Tyr128@OH**	6.50	0.39	6.50
**Ser198@Oγ**	**His438@NE2**	3.83	0.26	2.60
**Tyr332@OH**	**Asp70@OD2**	4.37	1.04	2.80
**Asp70@OD2**	**Trp82@N**	9.78	1.11	7.30

Table S2. Individual energies terms of MM/GBSA results with different parameters and models. (All data is expressed as mean and standard deviation (M ± SD) in units of kcal/mol.)

Component	Energy	Component	Energy
ΔG_{vdW}	-26.27 ± 1.77	ΔG_{ele}	-5.79 ± 1.38
ΔG_{SA}	-3.19 ± 0.14	ΔG_{GB}	18.36 ± 1.68
ΔG_{nonpol}	-32.06 ± 2.41	ΔG_{pol}	15.16 ± 1.60
		ΔTotal	-16.90 ± 1.61

Table S3. The mean and standard deviation (M ± SD) of binding free energy for mutational complexes. Energies are in kcal/mol.

	**Trp82Ala**	**Pro285Ala**	**Phe329Ala**	**His438Ala**
$\boldsymbol{G}_{\mathbf{vdW}}$	-22.21 ± 1.31	-25.67 ± 1.78	-24.38 ± 1.77	-25.08 ± 1.76
$\boldsymbol{G}_{\mathbf{ele}}$	-4.96 ± 1.35	-5.77 ± 1.40	-5.42 ± 1.40	-5.32 ± 1.39
$\boldsymbol{G}_{\mathbf{GB}}$	16.52 ± 1.48	17.25 ± 1.81	16.96 ± 1.74	16.96 ± 1.74
$\boldsymbol{G}_{\mathbf{surf}}$	-2.84 ± 0.14	-3.13 ± 0.14	-3.00 ± 0.13	-3.11 ± 0.15
$\boldsymbol{G}_{\mathbf{gas}}$	-27.17 ± 1.81	-31.44 ± 2.39	-29.81 ± 2.43	-30.41 ± 2.30
$\mathbf{G}_{\mathbf{solv}}$	13.69 ± 1.42	14.12 ± 1.74	13.96 ± 1.68	13.85 ± 1.68
$\Delta\boldsymbol{G}_{\boldsymbol{binding}}$	-13.48 ± 1.37	-17.32 ± 1.67	-15.85 ± 1.73	-16.55 ± 1.63
